# Energy-Efficient Wireless Sensor Network with an Unequal Clustering Protocol Based on a Balanced Energy Method (EEUCB)

**DOI:** 10.3390/s21030784

**Published:** 2021-01-25

**Authors:** Ahmed A. Jasim, Mohd Yamani Idna Idris, Saaidal Razalli Bin Azzuhri, Noor Riyadh Issa, Muhammad Towfiqur Rahman, Muhammad Farris b Khyasudeen

**Affiliations:** 1 Department of Computer System and Technology, Faculty of Computer Science and Information Technology, University of Malaya, Kuala Lumpur 50603, Malaysia; ahmed.jasim@siswa.um.edu.my (A.A.J.); saaidal@um.edu.my (S.R.B.A.); issanoor82@gmail.com (N.R.I.); 2 Center for Research in Mobile Cloud Computing, University of Malaya, Kuala Lumpur 50603, Malaysia; 3 Electrical and Computer Systems Engineering, School of Engineering, Monash University Malaysia, Subang Jaya 47500, Malaysia; Muhammad.rahman@monash.edu; 4 Faculty of Electrical Engineering, University Technology MARA (UiTM), Shah Alam 40450, Malaysia; farris4317@uitm.edu.my

**Keywords:** wireless sensor networks, unequal clustering, energy consumption, network lifetime

## Abstract

A hot spot problem is a problem where cluster nodes near to the base station (BS) tend to drain their energy much faster than other nodes due to the need to perform more communication. Unequal clustering methods such as unequal clustering routing (UDCH) and energy-efficient fuzzy logic for unequal clustering (EEFUC) have been proposed to address this problem. However, these methods only concentrate on utilizing residual energy and the distance of sensor nodes to the base station, while limited attention is given to enhancing the data transmission process. Therefore, this paper proposes an energy-efficient unequal clustering scheme based on a balanced energy method (EEUCB) that utilizes minimum and maximum distance to reduce energy wastage. Apart from that, the proposed EEUCB also utilizes the maximum capacity of node energy and double cluster head technique with a sleep-awake mechanism. Furthermore, EEUCB has devised a clustering rotation strategy based on two sub-phases, namely intra- and inter-clustering techniques, that considers the average energy threshold, average distance threshold, and BS layering node. The performance of the proposed EEUCB protocol is then compared with various prior techniques. From the result, it can be observed that the proposed EEUCB protocol shows lifetime improvements of 57.75%, 19.63%, 14.7%, and 13.06% against low-energy adaptive clustering hierarchy (LEACH), factor-based LEACH FLEACH, EEFUC, and UDCH, respectively.

## 1. Introduction

**Figure 1:**
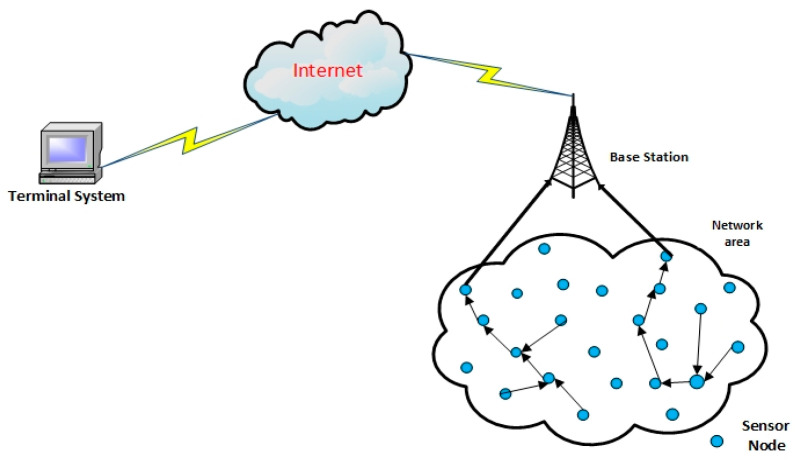
Wireless sensor networks (WSNs) architecture.

Wireless sensor networks (WSNs) comprise a large number of sensor nodes and sub-nodes with a restricted battery power supply. Generally, the sensor nodes are randomly distributed in the monitoring region to aggregate the collected data and transmit information to the base station (BS) or sink node by a single-hop or multi-hop [1,2,3]. The data are transferred to the terminal system through a communication link such as a satellite or the internet by the BS [4,5]. In the final stage, users will collect the data from the terminal system and manage the operation through it. The architecture of WSNs is shown in Figure 1. However, there are a few disadvantages in using WSNs, including a shorter network lifetime, higher energy consumption, instability, complicated network management if run on a large scale, and additional network overheads [6,7]. Due to their low-cost implementation, WSNs are widely employed in various applications such as industry, transportation, agriculture, the medical industry, environmental monitoring, and smart home systems [8] etc. However, the battery-powered sensor node has limited energy, and a complicated battery might change procedure, which affects the quality, performance, and lifetime of WSNs. Due to these factors, controlling energy consumption is one of the major problems concerning WSNs. An important fact to note when attempting to reduce this problem is also that data transmission of wireless communication consumes more energy compared to data processing. 

In the original methods, the initial idea was for the sensor nodes to collect the data and channel it directly to the BS node. When the process occurs, it leads to long-distance communication and results in higher energy consumption. Traffic and collisions will occur when sensors are transmitting data within the same period of time; this scenario will also result in data re-transmission and higher energy consumption. In order to solve this and to increase network lifetime, a reconsideration of routing and clustering [9,10] is required. In a clustering mechanism, the sensor nodes are divided into several clusters. Each cluster contains a central cluster known as the cluster head (CH) and several nodes of the cluster called cluster members (CM) [11,12,13]. The CMs receive data from an environment and send them to the CH. Once the CH receives the data, it will aggregate them in order to avoid data redundancy; the data will then be sent over to the BS via single-hop or multi-hop [14,15]. The clustering method has many advantages, including balanced energy consumption, scalability, and an improved network lifetime. The benefits and classifications of the clustering method have been widely described and defined [7,16,17]. However, the clustering protocols have some problems, such as transmission data to the sink, sometimes increasing energy consumption in WSNs [18] because the farthest distance CH will consume more energy to send data to BS. Multi-hop communication is usually adopted to save energy [19]. 

However, in the multi-hop communication method, the CH, which is closer to the BS, will take on more forwarding tasks. This will result in a massive overhead of the CH, and these CHs will run out of power sooner than the others. This causes a breakdown of the cluster and a loss of communication between CHs in a breakdown called the hot spots problem. Furthermore, as the CH is responsible for the aggregating and transmission process, when the CH receives data from the CM, the CH will aggregate data and forward them to the BS. As the CHs consume more energy than the CM, this also leads to unbalanced energy distribution in the overall network. If the CHs are not in close proximity, the furthest one from the BS node will dissipate more energy and increase the overhead.

In addition, the clustering protocols are divided into “static, dynamic, and hybrid” classes. In static clustering, clusters remain the same throughout the network’s lifetime once they are formed. The advantage of this method is that the overhead of clustering will be reduced. However, the static method may not perform correctly as some nodes may lose their energy and be terminated earlier than the other nodes. By contrast, dynamic clustering differs from the static method in that it will perform new clustering at each instance. Nevertheless, it has the disadvantage of high overhead, but it will not result in traffic that will pressure the nodes. Lastly, hybrid clustering improves energy consumption and network lifetime while reducing the communication overhead [20]. 

The clustering size approach can be classified into equal and unequal size clusters. In equal clustering, the size of the clusters is consistent throughout the network. Conversely, in unequal clustering, the size of clusters differs throughout the network based on the distance to the BS [21].

The hot spots problem arises when the cluster nodes are located closer to the BS node, where it takes more energy to receive data and forward it to the BS or server. Some of the possible solutions include using an unequal clustering-based routing in WSN, such as an energy-efficient unequal clustering routing (UDCH) and energy-efficient fuzzy logic for unequal clustering (EEFUC) protocols. These protocols are proposed in order to solve the hot spots problem and reduce energy consumption. However, these schemes do not utilize minimum and maximum distance to reduce energy wastage, and not much attention is given to enhance the data transmission in the network, which can lead to energy wastage across the network and a reduced network lifetime [4]. 

Moreover, these schemes do not have a sleep-awake mechanism, which can lead to network lifetime reduction. UDCH proposes an unequal clustering mechanism based on the competition radius. The calculation of the competition radius for each node depends on the residual energy of sensor nodes and the distance from the cluster nodes to the BS; however, it does not consider the fact that the minimum distance is the closest distance of a node from the BS, and that the maximum distance is the farthest distance of the node from the BS, which leads to an increase in energy consumption. Moreover, UDCH proposes a double cluster head in order to reduce the load on the primary CH. The selection process of the primary CH is determined based on computing the delay time of each node. The second CH is determined based on the distance from the sensor nodes to the primary CH. 

However, in UDCH, not much attention is given to enhance the data transmission process between sensor nodes and CH nodes in the network. To enhance data transmission among sensor nodes, the factor-based LEACH (FLEACH) is proposed. FLEACH avoids the selection of secondary cluster head (2CH) with low residual energy to achieve load balance between CH nodes. However, FLEACH does not address the hot spots problem [18]. The FLEACH protocol proposes a double cluster head in order to reduce energy consumption and prolong the network lifetime. The primary CH is randomly and alternately selected among the network nodes based on probability, which leads to an increase in energy consumption and the selection of 2CHs based on the highest residual energy of nodes. 

This paper proposes an energy-efficient routing protocol with an unequal clustering scheme based on a balanced energy method (EEUCB). The EEUCB protocol is focused on improving energy consumption, network lifetime and solving the hot spots problem by adopting unequal clustering technology. The size of the cluster depends on the distance between the cluster nodes and the BS. The CH that is closer to the BS nodes takes more energy to receive data and forward them to the BS. To resolve this problem, the EEUCB protocol is proposed to reduce the size of the CH, thus reducing its overhead. Meanwhile, EEUCB considers the energy of the CH as another metric in cluster size decisions, besides the distance. The CHs with more residual energy will form more massive clusters. 

On the other hand, we propose a double cluster head to reduce the overhead and energy consumption of the CH node. In this method, each cluster has two CHs. The primary CH is responsible for aggregating and forwarding data to the BS node if its distance is greater than the distance threshold and the energy consumption is less than the energy threshold. The 2CH is responsible for receiving and aggregating data within each cluster and sending them to the primary CH if the distance of the 2CH is less than the distance threshold and the energy is greater than the energy threshold. EEUCB proposes a different election mechanism for both the CH and 2CH. Calculating the delay-time for each CH node improves the election mechanism of the primary CH. As for the 2CH election mechanism, we consider the highest residual energy and the distance between nodes and the BS. The minimum distance between non-CHs and CHs can reduce the delay, improve energy consumption, and reduce transmission time. 

To balance the energy consumption among CMs and the CH, a clustering rotation strategy based on the average energy threshold, average distance threshold, and performance of layering by the BS node is proposed; this can increase network lifetime and the efficiency of energy consumption. Two techniques are proposed for transmission: (i) intra-cluster transmission for head clusters to share the data between them; and (ii) the inter-clustering transmission is proposed when the CH is placed at a great distance from the BS. These two techniques will reduce the overhead and avoid delays in the network. The contributions of this paper are as follows:

- We propose an energy-efficient routing protocol with an unequal clustering scheme based on the balanced energy EEUCB method.
- We improve the selection of 2CH to reduce the load and overhead on the primary cluster head by calculating the highest residual energy.
- To preserve the energy, we apply the sleep-awake mechanism based on the distance from sensor nodes to CH and the energy levels of sensor nodes to reduce energy consumption and prolong the network lifetime.
- To avoid the long distances among nodes, we propose distributing the nodes depending on the divided network layers by calculating the furthest and closest node to the base station.
- We enhance the transmission round between CMs, CHs, and BS by utilizing the average distance threshold and average energy threshold to prolong the lifetime of nodes in the network via intra-cluster transmission. Meanwhile, it utilizes the layer implementation and residual energy of clusters to construct a path to BS via inter-cluster transmission.

The rest of this study is organized as follows. Section 2 presents a number of routing protocols proposed for WSNs to be described in section related work. Section 3 and Section 4 describe the methodology of the proposed approach and evaluation metrics. Section 5 and Section 6 present the simulation setup, results, and discussion. Finally, Section 7 summarizes this study and outlines future work. 

## 2. Related Work 

**Figure 2:**
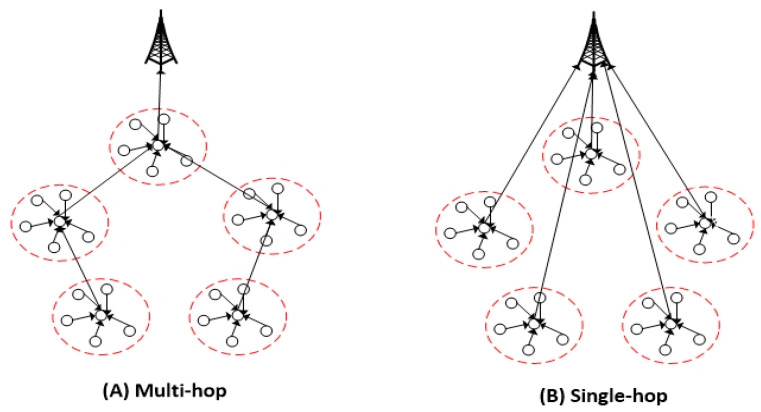
The structure of the clustering routing protocol.

The previous works proposed a clustering routing protocol for wireless sensor networks. The clustering routing protocol is proposed to save energy consumption and prolong the network lifetime. The clustering is a typical hierarchical routing protocol. The main idea of clustering is for the whole network to be divided into small sets of nodes and be distributed in the network called “cluster.” The structure of a network based on the clustering routing protocol, such as single-hop and multi-hop, is shown in Figure 2. Many researchers have proposed protocols based on clustering algorithms to improve energy consumption and prolong the network lifetime. The low-energy adaptive clustering hierarchy (LEACH) was proposed in [22]. In this protocol, cluster heads are randomly selected based on a threshold, and the neighbor nodes of the cluster head are selected by the nearest cluster heads to form cluster members. This protocol combines data fusion with a routing protocol to reduce the amount of data that needs to be transmitted to the base station. However, this method does not address the hot spots problem, and the random selection cluster head leads to loss of energy and terminates sooner. Many protocols are proposed to improve the LEACH protocol, such as hybrid energy-efficient distributed clustering (HEED). This protocol is the most well-known clustering algorithm in WSNs [23]. This algorithm employs hybrid parameters for the election of CH, which depends on node energy and communication cost. Nodes with higher energy have a higher chance of being selected as CH. Communication cost might depend on a node degree or the inverse of a node degree. However, this algorithm has a higher overhead because clustering is performed dynamically. 

To reduce the overhead, a low-energy adaptive tier clustering hierarchical routing (LEATCH) protocol is proposed. This protocol consists of a two-tier hierarchical clustering approach. The clusters are divided into mini clusters. Each mini-cluster communicates with the cluster head node to distribute the operations among cluster nodes [24]. In order to modify CHs selection [25], a modified low-energy adaptive clustering hierarchy (LEACH-M) is proposed. The main idea of this method is to optimize CHs selection and balanced energy consumption based on both the distributed residual energy and location addresses of nodes to optimize the CH threshold equation. On the other hand, a distributed energy-efficient clustering (DEEC) protocol is proposed to elect the cluster head node used a ratio of residual energy of a node and the average energy of the network. However, the location of cluster nodes that close to the BS was not considered in the prior algorithm. This will lead to the hot spots problem, where nodes will utilize more energy to receive and transmit data in a network [26]. 

Several methods have been proposed to solve this problem. One of these is an energy-efficient unequal clustering mechanism (EEUC) for data aggregation application in a wireless sensor network. This protocol utilizes localized computation for the selected cluster head. The advantage of this protocol is that it divides the network into unequal size clustering, which balances the energy consumption in the network. Clusters closer to the BS node are smaller in size than those far from the BS [27]. However, this protocol utilizes one cluster head for aggregation and data transmission to the base station [28]. The energy-efficient multi-hop unequal clustering method was proposed based on the routing approach. The advantage of this protocol is that the selection of CHs was based on residual energy of sensor nodes and used the Dijkstra routing algorithm to create the shortest path between the nodes and CHs. An improved energy-aware distributed unequal clustering protocol (EADUC) was proposed to improve CHs selection and solve the hot spots problem [29]. The advantage of this method was addressed to the hot spots problem and the election of CH via information and numbering of nodes. In addition, a fuzzy energy-aware unequal clustering algorithm (EAUCF) protocol was proposed by [30]. This protocol is proposed in order to solve the hot spots problem and reduce the energy consumption in the network. The method proposes a probabilistic model for the selection of CH. However, balanced energy consumption among nodes was not considered [31]. The balanced-imbalanced cluster algorithm (B-IBCA) is proposed. This method was proposed to reduce energy consumption and prolong the network lifetime based on the stabilized Boltzmann approach. The advantage of this method, the selection of CH is based on the distance and residual energy consumption in the network. However, this method has not reduced the load on the cluster head node and was not applied to the sleep-awake mechanism, which leads to an increase in energy consumption in the network. For a similar problem, there is a two-tier distributed fuzzy logic-based protocol (TTDFP). This method was proposed to increase network lifetime based on a fuzzy logic approach [32]. The advantage of this method addressed the hot spots problem and utilized the distance to the base station for the selection of CH. However, the double cluster head and sleep-awake mechanism were not addressed. Therefore, this method increases energy consumption for receiving and transmitting data to BS. Reference [33] proposed an energy-efficient cluster head selection scheme (EECHS). However, this CH method is randomly and alternately selected among the network nodes based on probability.

In addition, fuzzy-based unequal clustering (FBUC) is proposed [15]. The fuzzy system algorithm is used to determine the radius of nodes with inputs of residual energy and a number of neighbor nodes. The advantage of this protocol is that it reduces the transmission delay. However, the overhead is increased by this method in a way that leads to a reduction of network lifetime. To solve this problem, one solution proposed is energy conserved unequal clusters with fuzzy logic (ECUCF) in order to solve the hot spots problem and reduce energy consumption. The selection of CH is randomly based on probability between nodes, and the base station is randomized rotationally [34]. However, this method utilizes one cluster head for aggregation and data transmission to the base station [35]. A distributed clustering algorithm guided by the base station (DCAGBS). This method proposed a distributed approach to form the clusters dynamically to span the network lifetime. However, with this method, the hot spots problem was not addressed [36]. A multi- clustering algorithm using fuzzy logic (MCFL) is proposed. This method proposed a fuzzy logic for selecting multi-clustering algorithm nodes that are clustered in different rounds using different algorithms. The specific round for elect CHs was reduced overhead. To enhance the performance of the MCFL method, an energy-efficient fuzzy logic for unequal clustering (EEFUC) protocol is proposed to enhance the MCFL protocol. This protocol was proposed by [19] to reduce the energy consumption in the network by multi-hop clustering using the fuzzy logic method. 

However, balanced energy consumption was not considered. In addition, a multi-objective fuzzy clustering algorithm (MOFCA) was proposed to solve the hot spots problem and energy hole problem [37]. This method balances energy consumption and the selection of CH based on energy competition among nodes. However, this method non considers the double cluster head and sleep-awake mechanism. To solve these problems, the energy-efficient unequal clustering routing protocol for wireless sensor networks (UDCH) exists. This protocol was proposed by [4] to reduce the energy consumption of head nodes in the cluster and to solve the hot spots problem. However, the balanced energy consumption using an energy threshold only without addressing the distance threshold, which means that the distance among nodes farthest from CH or 2CH will lead to reduction of the network lifetime and increase energy consumption. The impact of the secondary cluster aggregation based on location in WSNs (FLEACH) was proposed in [18]. The proposed protocol employs the second cluster to aggregate the data and send it to the primary CH based on the location threshold and energy threshold to reduce energy consumption by reducing overhead and increasing network lifetime. However, this protocol does not address the hot spots problem. Also, randomly selected CH similar LEACH protocol processing leads to the loss of more energy for selection, in which case the nodes will soon die. Table 1 summarizes existing related works. Table 10 (see the conclusion Section 7) shows a comparison between our method and prior methods. 

Several methods and protocols designed to reduce energy consumption and prolong network lifetime in WSNs were reviewed. Different approaches were employed by the clustering protocol, including but not limited to the selection of CHs, double cluster heads, CH rotation, and redundant data transmission. However, in all these studies, balancing energy consumption and network lifetime still remains challenging due to the poor balance in energy consumption, the distance among nodes, and the load transmission on the CH node in the network. The novelty of this research consists in our ability to enhance the balanced energy consumption and to reduce the load transmission on the CH node in the network. The LEACH, FLEACH, EEFUC, and UDCH protocols were chosen to measure the reliability of our proposed EEUCB protocol. The LEACH protocol was chosen due to its common energy consumption radio model with our protocol. The FLEACH protocol was also chosen due to its common selection of the 2CH with our protocol to reduce the load on the primary CH. The EEFUC was chosen due to the selection of multi-hop clustering. Lastly, the UDCH was chosen in consideration of the hot spots problem and due to its common selection of the primary CH with our protocol in WSNs. 

## 3. The Proposed Energy-Efficient Unequal Clustering Scheme Based on a Balanced Energy Method (EEUCB) Protocol 

This section presents the details of our proposed energy-efficient routing protocol with an unequal clustering scheme based on a balanced energy method (EEUCB) for WSN. Before explaining the details of our protocol, we will first describe the network model and the energy model in the following sub-sections. 

### 3.1. Network Model 

In this sub-section, sensor nodes are randomly distributed in an environment of M*M square area, BS node has unlimited power, and outside of the monitoring area, each node can be adjusted as a CM or a CH. All nodes and BS are stationary and can adjust transmission power in order to estimate the distance and communication range; each sensor node has limited power, a unique ID, and is aware of each of the locations in the network. Each cluster has two CHs, the primary cluster head CH and the second cluster head 2CH. The primary CH is responsible for receiving data from nodes and sending multi-hop to BS, while 2CH is responsible for receiving data from nodes, aggregating the data, and sending it to primary CH. The energy consumption of setting up the network can be described as follows:

1. The BS sends a broadcast Hello message to all sensor nodes.
2. Each sensor node calculates its distance to the BS based on the signal strength indicator (RSSI) mechanism.
3. The intermediate neighbor nodes that forward information to other intermediate neighbor nodes until they reach the BS.
4. The BS then calculates the distance difference among the sensor nodes from the BS and empirically divides it into four layers to transmit data from the CHs to the BS.
5. The sensor nodes can send data to the CHs based on the distance and energy thresholds.
6. The CHs forward the data aggregated to the BS via single or multi-hops based on the location of CHs in the network.

### 3.2. Energy Model 

To evaluate the performance of the proposed EEUCB scheme, we use the energy model that is similarly described in the LEACH protocol [22]. The communication between nodes consumes the vast majority of energy, so the energy consumption is neglected for sensing and processing in this work. In the process of communication, the transmission and receiving processes consume more energy than monitoring. Therefore, we consider the energy of transmitting and the energy of receiving as energy consumption for communication. The energy consumed by transmitting l-bit data over distance d meters can be calculated as:

(1) $$E_{TX}=\;\{\begin{array}{c} k.E_{elec}+k.efs\times d^{2}\;\;\;\;\;\;\;when\;d\leq \;d_{0}\\ k.E_{elec}+k.emp\times d^{4}\;\;\;\;\;when\;d\geq d_{0} \end{array}\;\;\;\;\;\;\;$$

where $\;E_{TX}$ is the energy consumption of transmitter, k is the length of transmission data, $E_{elec}$ is the energy consumption of the receiver circuit or sender circuit for 1-bit data, efs is the data energy consumption of 1-bit in free space mode ($d^{2}\;\mathrm{power}\;\mathrm{loss}$), emp is the data energy consumption of a 1-bit in multi-path attention mode ($d^{4}$ power loss), d is the communication distance, and $d_{0}$ is the threshold distance value, if the node distance is less than the distance threshold, it will send data via free space, while if the node distance is greater than the distance threshold, it will send data via multi-path to avoid the high energy consumption during the transmission or receiving data. The threshold distance value $d_{0}$ can be calculated as:

(2) $$d_{0}=\sqrt{\frac{efs}{emp}}$$

Moreover, to receive this message, the radio expends energy:

(3) $$E_{RX}\left(i\right)=k.E_{elec\;\;}$$

where $E_{RX}$ is the energy consumption of the receiver, and $E_{elec}$ is the energy consumption of receiver circuit or sender circuit for 1-bit data.

### 3.3. Proposed EEUCB Protocol 

The hot spots problem is one of the most critical challenges in WSNs. It is the primary reason for unbalanced energy consumption when multi-hop transmission between clusters with BS is performed. CHs closest to the BS will consume more energy than other CHs due to the receiving of more forwarding tasks. In order to solve this problem, EEUCB is proposed. Our goal is to reduce energy consumption in order to increase the network lifetime. In EEUCB, cluster member nodes have different sizes and different regions. The sizes of CHs will be smaller than others if the cluster nodes are closer to the BS. This can eventually help us to avoid the node dying early when more than CHs share the data relay task in these areas. Moreover, the sleep-awake mechanism is utilized in our protocol based on the distance range from sensor nodes to CH as well as to the energy level, so it will prolong the network lifetime and preserve energy consumption. Secondly, in order to reduce the load and responsibility of CHs, the secondary CH (2CH) mechanism is proposed. The primary CH is responsible for forwarding data between the clusters, and 2CH is responsible for receiving and aggregating data within each cluster. 

**Figure 3:**
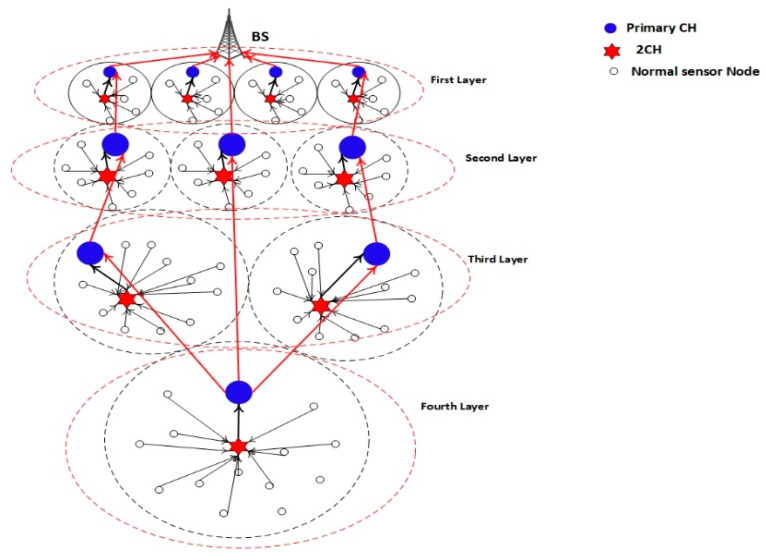
The network system design.

To balance the energy consumption among CMs and the CH, a clustering rotation strategy based on the average energy threshold, average distance threshold, and performance of layering by the BS node is proposed. The network system architecture design based on the EEUCB protocol is shown in Figure 3. 

**Figure 4:**
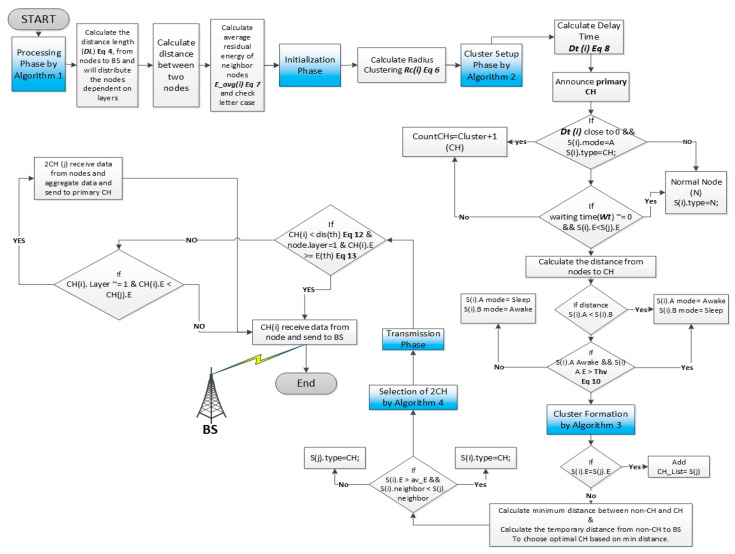
Flowchart of energy-efficient unequal clustering scheme based on a balanced energy method (EEUCB) protocol.

The EEUCB protocol contains four phases: the processing phase, initialization phase, cluster setup phase, and transmission phase. The processing phase is responsible for estimating the distance length with the base station node and calculating the average residual energy of neighbor nodes. The responsibility of the initialization phase is to calculate the radius of clustering to generate unequal cluster in the network. The cluster setup phase is responsible for calculating the delay time for the election of the primary cluster head node. Lastly, the transmission phase is responsible for transmitting data between the cluster member and base station node and balancing energy consumption between the sensor nodes and cluster head nodes. These phases are explained in the following sections. To better understand the protocol, Figure 4 shows the EEUCB flowchart. The detail of notations and descriptions can be found in Table 2. 

#### 3.3.1. Processing Phase 

The processing phase function estimates the distance between cluster nodes and neighbor nodes and checks the number of neighbor nodes. First, after the sensor nodes are deployed in the network, the BS broadcasts a message in the network, after which each sensor node calculates its distance to the BS based on the RSSI mechanism to identify the location of the sensor nodes [38]. The sensor nodes then send their location information to the BS. In this case, to be more realistic, our proposed method does not assume that all the sensor nodes can reach the BS through their own signal strength. Instead, we utilized intermediate neighbor nodes that forward information to other intermediate neighbor nodes until they reach the BS. The BS then calculates the distance difference among the sensor nodes from the BS and empirically divides it into four layers to transmit data from the cluster head to the BS. In this research, we do not focus on the number of layers, but the goal of these four layers is to identify the distance of each node from the BS and help the multi-hop communication between clusters to reduce energy consumption [38]. 

In addition, the place of cluster nodes is distributed in the network based on the layering mechanism. After dividing the sensor nodes into various layers according to their distance from the BS, the BS notifies each node as to which layer it belongs to. In our method, four layers, as shown in Figure 3, are proposed. The nodes are in the first layer (which is close to the BS), and the data is transmitted to the BS in a single hop. On the other hand, nodes in the second, third, and fourth layers utilize a multi-hop mechanism. The first layer has the smallest cluster size (with more clusters), followed by the second, third, and finally the fourth with the greatest cluster size (with fewer clusters). Unlike the prior equal clustering method, the relay tasks in the proposed method can be shared among cluster heads within the same layer. Nodes closer to the sink tend to drain their energy at a faster rate when compared to other nodes as they have to perform more communication. Therefore, sharing relay tasks can avoid a single cluster head needing to handle all incoming data from the higher layer (i.e., the hot spots problem). Besides this, the sleep-awake mechanism is also utilized in our proposed protocol. This mechanism relies on two criteria, namely the distance from the sensor nodes to the CH and the energy level. 

To make the BS determine the clustering strategy and perform layering, each node calculates the distance length of each layer. The distance length $d_{L}\;$ can be calculated as:

(4) $$d_{L}=\left(d_{max}-d_{min}\right)/4$$

where $d_{max}$ is the farthest distance of the node from the BS, and $d_{min}$ is the closest distance of a node from the BS. The clustering is performed independently by each of the layerings. Equation (4) improved to avoid the long-distance, so this will lead to an extension of the network’s lifetime and improvement of network stability. In addition, we propose an unequal clustering routing protocol; therefore, the location of the BS node will be outside the sensing area as described by [18]. If the location of the BS is inside the sensing area, the network lifetime will increase because more nodes will share the high energy consumption around BS. Therefore, we located the BS outside the sensing area to verify the network lifetime of our protocol. On the other hand, if the location of the base station is inside the sensing area and the sensor nodes are opposite the base station, the Pythagorean theorem can be used to verify the difference between the farthest and closest distance of nodes from the BS. Therefore, in this case, the BS will distribute nodes in the network based on the competition radius ($R_{c}\left(i\right))$ such as in Equation (6), if the $R_{c}\left(i\right)$ is greater than the maximum competition radius $RL_{max}$, the location of nodes will be either in the second, third, or fourth layer in the network. The clustering layers algorithm is described in Algorithm 1. The input and output parameters (Node(i), $d_{min}$, $d_{max}$, BS, $d_{L})$ of the algorithm are specified in 1–2. The sending and receiving of broadcast messages between nodes and BS are specified in steps 3–5. Step 6 calculates the distance length $\left(d_{L}\right)$. Each node evaluates the layer with the BS in step 7. In steps 8–17, each node compares the layer depending on the maximum and minimum distance with BS.

**Algorithm 1** The Clustering Layers of EEUCB1. Input (Node(i), $d_{min}$, $d_{max}$, BS)2. Output (Node(i). layers) 3. BS broadcast message to all nodes 4. Nodes receive a message from BS 5. Nodes send a status message to BS6. Calculate the distance length according to Equation (4)7. **For** all of the nodes, BS **do**8.  **If** the distance node (i) to BS < $d_{min}$ + $d_{L}$
**then**9.      Node(i). layer = L1;10.    **Else**
11.    **If** the distance node (i) to BS > $d_{min}$ + $d_{L}$ && distance node (i) to BS < $d_{min}\;$+ (L2 × $d_{L}$) **then**12.     Node(i). layer = L2;13.   **Else**
14.   **If** the distance node (i) to BS > $d_{min}$ +(L2 × $d_{L}$) && distance node (i) to BS < $d_{min}$ +(L3 × $d_{L}$) **then**15.     Node(i). layer = L3;16.   **Else**17.     Node(i). layer = L4; 18.   **End**19. **End**

#### 3.3.2. Initialization Phase 

If the sensor nodes are closer to the BS and are forwarding more data to it, the node will be heavier and consume more energy. To balance out the energy in the whole network, the initialization phase proposes generating unequal clustering in the network and electing the main CH by calculating the delay time of the nodes. An unequal algorithm could balance the energy among the sensor nodes. Hence, the size of the cluster nodes should be smaller than others if the cluster nodes are closer to the BS. The initialization phase is divided into three parts, namely unequal clustering generation, competition radius calculation, and delay time. 

##### Unequal Clustering Generation and Clustering Competition Radius 

In this sub-phase, the generation of unequal clusters is based on the competition radius. The competition radius is responsible for generating unequal clustering for each node and determining the size of the cluster with a BS node. Existing unequal clustering, such as in UDCH, utilizes the residual energy of sensor nodes and the distance from all the sensor nodes to the BS node. However, this method does not consider the length of the distance between CMs and the BS node, which leads to energy wastage across the network nodes and a reduced network lifetime. Apart from residual energy and the distance from all sensor nodes to the BS as in the prior method, our proposed EEUCB method also considers the minimum distance of the closest node from the BS and the maximum distance of the furthest node from the BS. These two criteria were considered to avoid long-distance; this will extend network lifetime and improve network stability. Furthermore, our protocol utilizes the maximum capacity of node energy to improve the existing method. The calculation of the competition radius $\left(R_{c}\right)$ of the prior method (UDCH) is shown below:

(5) $$R_{c}\left(i\right)=a\frac{E_{rem}\left(i,r\right)}{E_{init}}+b\frac{d_{i,BS}}{D_{max}}\;RL_{max}$$

The calculation of competition radius $\left(R_{c}\right)$ of our proposed EEUCB protocol is shown below:

(6) $$R_{c}\left(i\right)=[1-a\left(\frac{D_{max}-d_{i,BS}}{d_{max}-d_{min}}\right)-b\left(1-\frac{E_{rem}\left(i,r\right)}{E_{max}}\right)]\;RL_{max}$$

where $R_{c}\left(i\right)$ is the radius of node i, $D_{max}$ is the maximum distance from nodes to BS, $d_{i,BS}$ is the distance from node i to BS, $E_{rem}\left(i,r\right)$ is the residual energy of node i at round r,$\;E_{init}$ is the initial energy of node i, $E_{max}$ is the maximum capacity of node energy,$\;RL_{max}$ is the maximum competition radius for becoming CH, and (*a*) and (*b*) is the weighted factor between [0,1] to adjust the scope of $R_{c}\left(i\right)$. Moreover, it determines the impact of the energy and distance on the competition radius. When (*a*) and (*b*) increases, the range of competition radius value decreases; conversely, when (*a*) and (*b*) decrease, the range of competition radius value increases, also including the effect of competition radius value on the energy. 

##### Delay Time 

**Figure 5:**
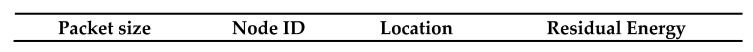
Format of data packet type_1.

After calculating the competition radius $R_{c}\left(i\right)$, the sensor nodes then calculate the delay time $D_{t}\left(i\right)$ to announce being a CH; the delay time is computed in Equation (8). The delay time scheme in this paper used timestamp synchronization. It provides internal synchronization by request. The calculation of the said process depends on the residual energy of neighbor nodes $E_{rem\left(j,r\right)}$, the number of neighbor nodes ⋮NN(i,r)⋮, and the average residual energy of neighbor nodes $E_{avg\left(i\right)}$ such as in Equation (7). Therefore, the sensor nodes will send a broadcast packet to the neighbors. The content of the packet includes the node ID, residual energy, location of the node, packet size, and the type of packet (packet type_1) because different types of broadcast packet are sent in the network to calculate the average energy of the neighbor nodes. Each node records the information of neighbor nodes when they receive the packet, and the nodes are identified by the neighbors; the nodes will then calculate the average energy of neighbor nodes as defined in [12]. The average energy of neighbor nodes can be calculated as:

(7) $$E_{avg\left(i\right)}=\frac{\sum^{\;}j\epsilon NN\left(i,r\right)E_{rem\left(j,r\right)}}{\max\left(\vdots NN\left(i,r\right)\vdots ,\sigma \right)}$$

where $E_{avg\left(i\right)}\;$ is the average energy of neighboring node i, NN(i,r) is the set of neighbor nodes, ⋮NN(i,r)⋮ is the number of neighbors, j is the neighbor node of node i, and σ is a tiny number which has no effect on the Equation (8) if the result becoming zero. When many nodes have the same number of neighbors, σ plays a role in obtaining a different number of neighbors for each node. The delay time of each node can be calculated as:

(8) $$D_{t}\left(i\right)=\left(1-\frac{E_{rem\left(i\right)}}{E_{avg\left(i\right)}}\right)*W_{t}+R_{v}$$

where $\;\;D_{t}\left(i\right)$ is the delay time of node i, $E_{rem\left(i\right)}$ is the residual energy of node i, $W_{t}$ is the competition time of primary CH, and $R_{v}$ is the random value. The random value can play a role in reducing communication conflicts when the nodes have the same residual energy. The format of the (packet type_1) is shown in Figure 5. 

#### 3.3.3. Cluster Setup Phase 

In this section, the clustering setup phase will elect the nodes to become CH; the election of CH depends on the delay time processing. This section contains three sub-sections: primary CH selection and sleep-awake mechanism, cluster formation, and secondary CH selection. 

##### Primary Cluster Head (CH) Selection and Sleep Awake Mechanism 

**Figure 6:**
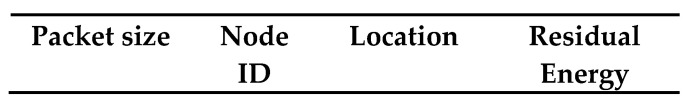
Format of data packet type_2.

The Primary CH selection method for the proposed EEUCB is similar to the UDCH [4]. However, in EEUCB, further improvement is made by taking into account the sleep-awake mechanism. The method starts with the calculation of the delay time, as in Equation (8). When the delay time closes in on zero, the primary CH will be elected. If a node is calculated to have a shorter delay time, it will have a higher chance to become a CH and send a broadcast packet. The content of the packet includes the packet size, node ID, location of the node, residual energy, and the type of packet (packet type_2). The format of the packet type_2 is shown in Figure 6. 

Each node should wait until the delay time process ends. If a node [i] receives packet type_2 from nodes before the delay time process ends, the node [i] will become normal nodes “N.” 

In addition, if the other nodes broadcast the packet type_2 and the candidate CH receives the packet before the end of the competition time $\;W_{t}$, it will compare the residual energy of node $\;S_{i}$, and node $\;S_{j}$. If the node $S_{i}$ is greater than node $\;S_{j}$, it will become primary CH; otherwise, it becomes a normal node “N.” Furthermore, the candidate CH will broadcast messages to sensor nodes. The sensor nodes will verify the messages from the CH. If the CH have limited residual energy and might die sooner, the nodes will inform neighbor nodes in the network to change the path and send data to another CH. 

**Figure 7:**
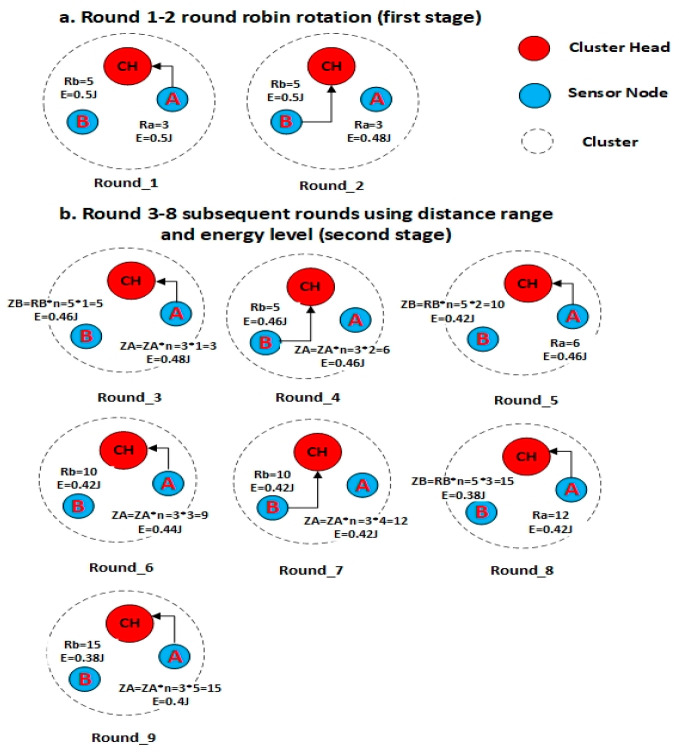
Sleep-awake rotation.

Since nodes with a shorter distance to the CH consume less energy, we propose a round-robin sleep-awake rotation method to select nodes to transmit data to the CH based on two stages. This method is illustrated in Figure 7. Let it be assumed that node A and node B are **3m** and **5m** away from the CH, respectively. In the first stage (Figure 7a), the round-robin-based selection depends only on the node distance to the CH. Since node A has the shortest distance to the CH, it is the first selected (awake) to transmit data to the CH (Round_1). The non-selected nodes will remain asleep. In the next round (Round_2), node B—which has the second shortest distance to the CH—is then selected to be awake and sends data to the CH. The process will continue until all nodes have transmitted their data to the CH. In the second stage (Figure 7b), the selection is not based on distance alone; the energy level becomes the additional criterion that is included in the rule. 

Apart from that, the utilization of distance, R, in the second stage, is modified based on Equation (9). Starting from Round_ 3, the distance, R, is used to calculate the variable Z as follows:

(9) Z= R∗n

where Z is the name of each node, R is the radius, and n is the variable increment if Z has the lowest value. 

From the example in Figure 7 (i.e., Round_3), node A is selected to be awake to transmit its data to the CH since it has the lowest Z value (i.e., ZA = RAx1 = 3 × 1 = 3). Node B (and others, if any), on the other hand, will remain asleep. Following the awake selection, the selected node A will then update its Z value (i.e., ZA = RAx2 = 3 × 2 = 6) in Round_4, while the Z value for the other nodes (which were not selected) remains as before. In round_4, the lowest Z value (i.e., Node B with ZB = RBx1 = 5 × 1 = 5) is selected to be awake once again. The selected node will update its Z value in the 5th Round; in this example, it can be seen that ZB is updated to ZB = 10 (i.e., ZB = RBx2 = 5 × 2 = 10). The process continues in the next round. 

Besides utilizing the Z value, the sleep-awake mechanism in the second stage also depends on the energy level according to the threshold value, as shown in Equation (10). Assuming that the $Th_{v}$ is equal to 0.02j, the current awake node—as long as it remains awake and continues to transmit data to the CH—should have residual energy greater than the threshold value. From the example in Figure 7 (i.e., Round_6), node A will remain awake and transmit its data to the CH since it has the lowest Z value and its residual energy is greater than the threshold value. Node B, on the other hand, will remain asleep. 

In addition, if the two nodes have the same Z value in the cluster, the node with the higher residual energy is chosen. From the example in Figure 7 (i.e., Round_9), nodes A and B have the same Z value, whereas node A will have higher residual energy than node B. Therefore, node A will remain awake and continue to transmit data to the CH, while node B remains asleep.

The threshold value of the node in the cluster is calculated as below:

(10) $$Th_{v}=\frac{T_{E}}{N}$$

where $T_{E}$ is the total energy of sensor nodes in the cluster, and N is the total number of sensor nodes. 

**Figure 8:**
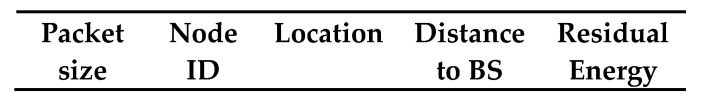
Format of data packet type_3.

After the sleep-awake mechanism, the primary CH will broadcast a message within the maximum competition radius $RL_{max}$ at the end of competition time $W_{t}$. It consists of a packet, which includes node ID, residual energy, location of the node, packet size, distance to BS, and the type of this packet (packet type_3). The format of the packet type_3 is shown in Figure 8. 

The primary CH algorithm is described in Algorithm 2. The input and output parameters ($S_{i},\;W_{t}$, A, B, $Th_{v}$, $RL_{max}$, *a, b,* $R_{v},\;R_{c}\left(i\right)$, $D_{t}\left(i\right)$, CH) of the algorithm is specified in 1–2. In steps 3–6, calculate the clustering radius $R_{c}\left(i\right)\;$ and broadcasting to all neighbor nodes. The number of neighbor nodes  NN(i,r)  and the average energy of neighbor nodes $E_{avg\left(i\right)}\;$ are calculated in steps 7–8. In step 9, the delay time is calculated for each node $\;D_{t}\left(i\right).$ In steps 10–20, the processing of nodes to select the primary CH is conducted, as explained in the above section. The residual energy between nodes in steps 21–30. The sleep-awake mechanism in steps 31–44. Finally, the primary CH broadcast type_3 packet within the competition time $W_{t}$ in steps 45–48. 

**Algorithm 2** Primary CH Selection and Sleep Awake Mechanism1. Input ($S_{i},\;W_{t},$ A, B, $Th_{v}$, $RL_{max}$, *a, b,*
$R_{v}$)2. Output (primary CH and mode)3. **For** each node $S_{i}$     **do**4. Calculate $R_{c}\left(i\right)$ according to Equation (6)5. Broadcast packet type_16. The nodes identified their neighbors7. Calculate the number of neighbor nodes     NN(i,r)8. Calculate the average energy of neighbor nodes $E_{avg\left(i\right)}$
9. Each node calculates the delay time $D_{t}\left(i\right)$ according to Equation (8)10.  $\;\;S_{i}.$ Type = “N.”11.   **If**
$S_{i}.$
$\;D_{t}\left(i\right)=$ close to 012.    CountCH= countCH +1 13.   $S_{i}.$ Type = “CH.”14.   Broadcast packet type_215.  **End**
16.  **While**
$S_{i}.$
$\;D_{t}\left(i\right)\neq$ close to   017.   **If**
$\;\;S_{i}$. $D_{t\;}>$
$S_{j}$. $D_{t\;}$
18.    $S_{i}.$ Type = “N.”19.   **End**
20.  **End**21. **While**   $S_{i}$. $W_{t}$
≠ 022.  **If**    $S_{i}$. $D_{t\;}$< $S_{j}$. $D_{t\;}$
23.   **If**  $S_{i}$. Type = “CH.”24.    **If** $S_{i}$. E < $S_{j}$. E25.     $S_{i}.$ Type = “N.”26.    **Else**
$S_{i}.$ Type = “CH.”27.    **End if**28.   **End if**29.   **End if**30.  **End while**31.   **If**
$\;\;S_{i}.$ Type = “CH.” 32.    **While** all nodes send data once to CH33.  Calculate the distance from nodes to CH34. **If**$\;\;S_{i}\left(A\right).\;\mathrm{distance}\;\mathrm{to}\;\mathrm{CH}\;<\;\;\;S_{i}\left(B\right).\;\mathrm{distance}\;\mathrm{to}\;\mathrm{CH}$
35.  $S_{i}\left(A\right).\;$ mode = “Awake.”36.  $S_{i}\left(B\right).$ mode = “Sleep.”37.  **End if**38.   **If** $S_{i}\left(A\right)\;Awake\;\&\&\;\;S_{i}\left(A\right).E>Th_{v}$
39.    $S_{i}\left(A\right).$ mode = “Awake.”40.    $S_{i}\left(B\right).$ mode = “Sleep.”41.  **Else**   $S_{i}\left(A\right).$ mode = “Sleep” **&**&      $S_{i}\left(B\right).$ mode = “Awake.”42.  **End if**
43.  **End while**
44. **Endif**45. **If**
$S_{i}.$ Type = “CH.” 46.   Broadcast packet type_3 to BS 47. **Endif**
48. **Endfor**

##### Cluster Formation 

The cluster formation function selects the optimal non-CH to become a member of the CH. After the primary CH selection in this sub-section, the CH will broadcast packet type_3 and the node $S_{i}$ as it awaits receipt of the packet. If the node $\;S_{i}\;\mathrm{receives}\;\mathrm{the}\;\mathrm{message}\;\mathrm{from}\;\mathrm{node}\;S_{j}$, it will add and store it to the list of candidate nodes for the CH (CH_list) and change it into a non-CH state. On the other hand, the node $S_{i}$ may possibly receive more messages from different CHs. In this case, it will choose the optimal nodes as its CH by calculating the distance from non-CHs to the CH. The optimal selection of the CH is dependent on the minimum distance, high residual energy, and a smaller number of neighbor nodes. In addition, the maximum number of nodes in each cluster will affect the performance of the network. Therefore, to balance the distribution of nodes among the cluster heads and to achieve balanced energy consumption, in this paper we define the maximum number of each cluster as calculated by $=\frac{N}{T_{CH}}$, where the N is the total number of sensor nodes, and $T_{CH}$ is the total number of CH in the network. At the time when sensor nodes join the cluster, the cluster head will compare the number of nodes with the t value. If the value is less than the t value, it will accept the node; otherwise, it will reject the request. 

Algorithm 3, the input and output parameters ($S_{i}$, $S_{j},\;\mathrm{CH}\_\mathrm{list})$ of the algorithm is specified in 1–2. Explains the procedure of cluster formation that chooses the optimal CHs in steps 3–11.

**Algorithm 3** Cluster Formation1. Input ($S_{i}$, $S_{j})$2. Output (CH_list)3. **For** each node $S_{i}$   **do**4. **If**   $S_{i}.$ Type = “N” && $S_{i}$. E > 0 && $S_{i}.$ Type = “Awake.”5.    **If**
$S_{i}.\;Head$ = $S_{j}.\;Head$6.        Compute the minimum distance from non-CH to CHs7.     **If** $\;\;S_{i}$. E > av_energy && $S_{i}$. Neighbor < $S_{j}$. Neighbor 8.       $S_{i}.$ Type = “CH.” 9.     Broadcast packet type_310.       CH_list store $S_{i}$11.     **Else if**
$S_{j}.$ Type = “CH.” 12.  **End if**13. **End if**14. **End for**

To reduce the overhead and processing load and to save energy by minimizing the energy consumption of primary CH, we propose to elect the secondary cluster head 2CH. The primary cluster head CH is responsible for aggregating data and forwarding it to the BS node if the primary CH distance is lower than the distance threshold, and the residual energy is greater than the energy threshold. Meanwhile, the 2CH is responsible for receiving and aggregating data within each cluster and sending the aggregated data to primary CH if the distance from the primary CH is greater than the distance threshold and the residual energy is less than the energy threshold. The Medium Access Control MAC algorithm between cluster members and CHs is based on time-division multiple access (TDMA). Whenever if the primary CH aggregates data from nodes, the primary CH creates the TDMA schedule for other normal nodes and broadcasts the schedule to all the cluster members. Otherwise, if the 2CH aggregates data from nodes, it will create the TDMA schedule for the normal nodes within the cluster. In this case, each node will send data to the 2CH based on the schedule. 

In Algorithm 4, we assume that CH is represented by ‘j’; and ‘f’ represented the sensor nodes distributed in the cluster. The input and output parameters of the algorithm are specified in 1–2. In steps 3–6, we define the cluster-ID as ‘j’ and the energy of non-CH as ‘f’ in per round in the network. To select the 2CH, our EEUCB protocol proposes that the sensor nodes with the highest residual energy $max_{E}$ will become 2CH. If the N(f) belongs to the cluster N(j) and the N(f) is equal to N(j), each cluster will compute the energy temporarily and find the maximum residual energy in each cluster as described in steps 7–15. Steps 16–19 will check the residual energy of N(f). If it is high, it will be selected as 2CH; otherwise, it will become a normal cluster node. 

**Algorithm 4** Selection of Secondary Cluster Head 2CH1. Input ( total number of nodes (n), the total number of CH ($n_{CH}$))2. Output (2CH)3. **for** each $n_{CH}$ to f  **do**4.    Cluster ID j = n (r + 1, f) 5.    Node ‘f ‘= E (r + 1, f) % energy per round 6. **End**
7.  **if**
$\;n_{CH}\;-1\geq$ 18.  **for** each cluster head j   **do**
9.    $max_{E}=0$10.   **if** N(f) ϵ j 11.    **if** N(f)= $n_{CH}\;\;$&& N(f). E > 012.     energy_temp= Max ($max_{E}$, E (r + 1, f)) 13.     **if** energy_temp > $max_{E}$14.      $max_{E}=$ energy_temp15.    **End**16.     **if** N(f). E == $max_{E}$17.       I = f    %% 2CH ID18.     **End**19.   **End if**20.  **End if**
21.  **End for**
22. **End if**


#### 3.3.4. Transmission Phase 

The transmission phase is the process of data transmission between CHs and CMs through the network. The transmission phase consists of a CH rotation strategy and a layering implementation. The CH rotation strategy and the layered implementation scheme will be further described in the next sub-section. The process of the transmission is utilized after the selection of the primary CH, which sends a broadcast schedule to all CMs by creating a TDMA schedule. The CMs will send data to the primary CH, which aggregates data. Thereafter, aggregation operations will be sent to the BS. 

##### CH Rotation Strategy and Layering Implementation 

The CH rotation strategy and layering implementation scheme are proposed in our EEUCB protocol to balance the energy consumption between CMs, CHs, and the BS nodes. We proposed this CH rotation strategy as the unbalanced energy consumption between sensor nodes and CHs during data transmission in the network will affect the network lifetime and increase energy consumption throughout the network. The rotation strategy functions to balance energy consumption between sensor nodes and CHs so that each node in the network has a chance to become a CH. 

A layering implementation is proposed in our EEUCB protocol in order to extend network lifetime and reduce energy consumption. The function of the layering implementation is to estimate the location of the primary CH in the network that was estimated on the basis of the distance to the BS. The layering implementation is performed, as shown in the processing phase Section 3.3.1.

Concerning the transmission phase of prior methods as in FLEACH and UDCH, the FLEACH utilizes average distance while UDCH utilizes the average energy threshold between CMs and CHs and constructs the path to the BS through the network. On the other hand, the proposed EEUCB utilizes both the average distance and energy threshold. The EEUCB also makes use of the layering implementation to reduce energy consumption and prolong the network lifetime. 

In our proposed EEUCB protocol, the CH rotation strategy includes two sub-phases, namely the intra and inter clustering transmission. In the first sub-phase, the CH performs the intra-cluster transmission. The CH rotation strategy is utilized between CMs and CHs. If the distance from the primary CH is less than the distance threshold, such as in Equation (12), and the residual energy is greater than the energy threshold, such as in Equation (13), the nodes will send data directly via single-hop to the primary CH. The primary CH then receives the data and applies aggregate functions. These nodes help the CH to consume less energy in receiving data. However, if the distance from the primary CH is greater than the distance threshold, and the residual energy is less than the energy threshold, the system will use multi-hop routing to transfer data to the CH, which consumes more energy. As such, the nodes will send data to the 2CH, which will aggregate data to be sent to the primary CH. Finally, the primary CH receives the aggregated data from the 2CH and sends it to the BS. In addition, if the distance is greater than the distance threshold and residual energy is greater than the energy threshold, the sensor nodes will send data to the 2CH. Conversely, if the distance is less than the distance threshold and the residual energy is less than the energy threshold, the sensor nodes will send data directly to primary CH. As the transmission process depends on distance, the transmission of data from CMs to CHs will not consume more energy in the network. 

**Figure 9:**
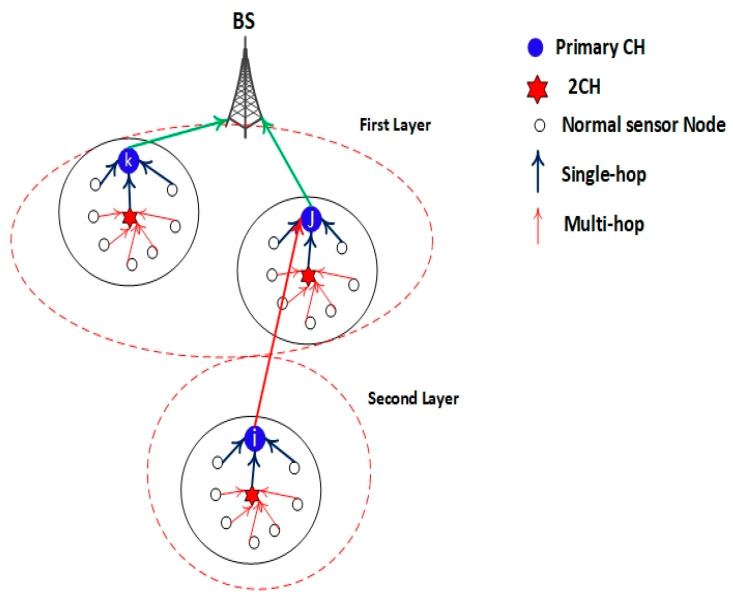
The intra- and inter-clustering transmission.

**Figure 10:**
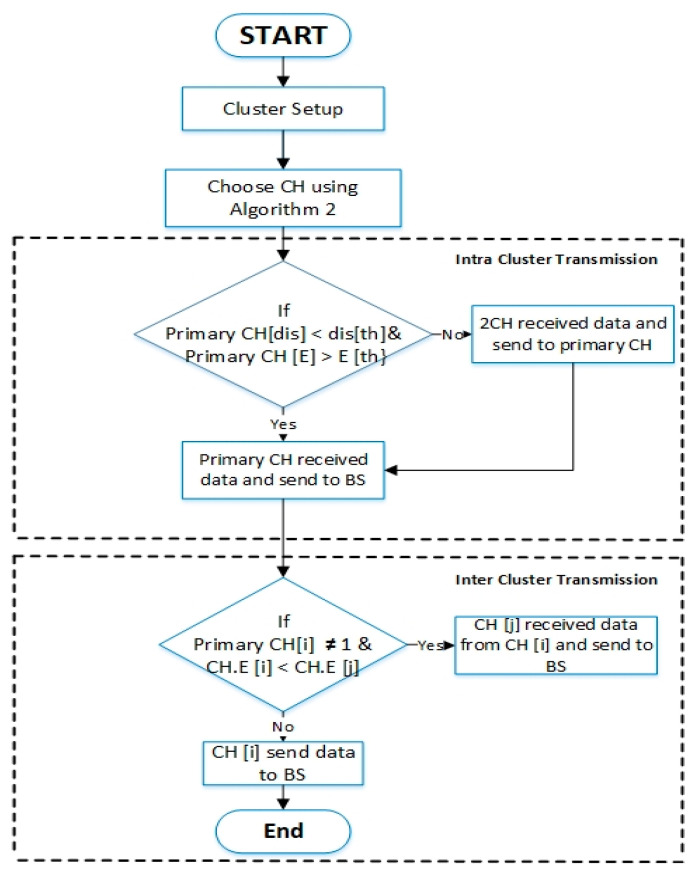
The flowchart of the main CH rotation strategy.

The second sub-phase is constructing a path to BS via inter-cluster layering. In this sub-phase, the MAC algorithm between the CHs and BS of our EEUCB protocol uses the carrier-sense multiple access (CSMA) for transmission data to BS. With the data transmission in this sub-phase, the sensor nodes might change their state into sleep mode. Therefore, the energy status and the number of node members will change in the cluster. Hence, if the CH [i] is not located in the first layer (not close to BS), and the residual energy of the CH [i] is less than the residual energy of CH [j], the CH [i] will send aggregated data to CH [j], and the CH [j] will transmit the data to BS; otherwise, the CH [i] transmit aggregated data to BS directly. The intra and inter clustering transmission based on the EEUCB protocol is shown in Figure 9. The flowchart of the main CH rotation strategy is shown in Figure 10. 

The distance threshold was considered because of the sensor node’s different distributions within the network area. So, if the primary CH distance is greater than the distance threshold, this means that the CH distance is farthest from the sensor nodes and will lead to more energy consumption in the data aggregation and transmission process. Therefore, the sensor nodes will send data to the 2CH, and the 2CH will then forward it to the primary CH. The distance threshold depends on the average distance. The average distance means the distance of all sensor nodes to BS, and the average distance can be written as:

(11) $$avg_{D}=\frac{1}{N}\overset{N}{\underset{i=1}{\sum }}D_{itoBS}$$

where $avg_{D}$ is the average distance, N is the set of sensor nodes, and $D_{itoBS}$ is the distance of each node to the BS. The Equation of the distance threshold can be calculated as:

(12) $$D_{th}=\partial \;*\;avg_{D}$$

where $D_{th}$ is the distance threshold and ∂ is the weight factor to determine the impact of the distance between the CMs and CHs. When the ∂ increases, the distance range of CHs is greater than the distance threshold. Hence, the 2CH will consume more energy for the receiving and transmission process, and data will not be aggregated. 

The energy threshold was also considered in our protocol in order to extend the network lifetime and to reduce energy consumption. When the energy of the primary CH is less than the energy threshold, the CH node will die early and disconnect the communication between nodes. To avoid this problem, we elect the 2CH to help the primary CH to be “alive” by receiving the data from nodes and executing aggregated functions, and after that sending it to the primary CH. The computation of the energy threshold can be written as follows:

(13) $$E_{th}=\;\beta \;*\;E_{CH}\left(i\right)$$

where $E_{th}$ is the energy threshold, $E_{CH}\left(i\right)$ is the initial energy of CH after each cluster selection, and β is the weight factor to determine the impact of the energy level on the energy threshold. 

## 4. Evaluation Metrics 

The proposed method has been presented in the previous section. To further test the reliability of the proposed method, four evaluation metrics are considered. These metrics measure the network lifetime, average energy consumption, average residual energy, and throughput. These metrics are explained in the following sub-sections. 

### 4.1. Network Lifetime 

The network lifetime is the time when the first sensor node in the network runs out of energy and dies. The measure network lifetime can be written as:

(14) $$\begin{array}{cc} N_{lifetime}=\overset{r\;max}{\underset{r=1}{\sum }} & \overset{N}{\underset{i=1}{\sum }}(Node^{\left(i\right)}.E\;\leq \;0,\;(dead\\ & =dead+1;\; \end{array}\;\;If\;\left(dead==1,\;first\_dead=r\right)))$$

where $N_{lifetime}$ is the network lifetime, r is the number of rounds, and dead=dead is the number of dead nodes around.

### 4.2. Average Energy Consumption 

Efficient energy consumption is significant in cluster network technology. One goal of our proposed protocol is to reduce and preserve energy consumption in the network. The average energy consumption can be calculated as:

(15) $$E_{avg}=\overset{N}{\underset{i}{\sum }}\;E_{ini}\left(i\right)/N$$

where $E_{avg}$ is the average energy consumption, $E_{ini}\left(i\right)$ is the initial energy of nodes (i), and N is the total number of sensor nodes.

### 4.3. Average Residual Energy 

The residual energy is energy within the system that is not being used but which, when released, can execute work. The residual energy is calculated when each sensor node is transmitting and receiving the packets among them in the network. The average residual energy can be computed as:

(16) $$E_{rem}=\;\overset{N}{\underset{i}{\sum }}\;E_{rem}\left(i\right)/N$$

where $E_{rem}$ is the average residual energy of nodes, $\;E_{rem}\left(i\right)$ is the remained energy of nodes (i), and N is the total number of sensor nodes.

### 4.4. End-to-End Delay 

The end-to-end delay is defined as the time taken when the packets transfer from the sensor nodes to the base station node. The end-to-end delay can be computed as:

(17) $$D=\overset{P\left(i\right)}{\underset{i+1}{\sum }}\frac{P_{t\left(i\right)}-P_{r\left(i\right)}}{Total\;packets}$$

where D is the end to end delay, $P_{t\left(i\right)}$ is the time when sending packets, and $P_{r\left(i\right)}$ is the time when the packets are received.

### 4.5. Throughput 

Throughput is the number of data packets successfully transmitted to the destination in a period of time. The formula of throughput can be written as:

(18) $$T_{p}=\frac{T_{s\;}}{T_{r}}sec$$

where $T_{p}$ is the throughput, $T_{s}$ is the total number of packets sent to BS and $T_{r}$ is the total number of packets received at BS. 

## 5. Simulation Setup and Results

In this section, our simulation is to compare the performance of EEUCB with other protocols using a MATLAB 2019b. The network topologies of this paper were presented by IEEE 802.15.4/ZigBee because this protocol supported clustering technology and carrier-sense multiple access (CSMA). On top of this, we want to investigate the energy consumption efficiency and network lifetime extension of our proposed protocol. Table 3 presents the three different scenarios, such as a different number of nodes and area sizes. All the examined scenarios show similar results regardless of node numbers and network area sizes. The parameters used in the simulation are presented in Table 4. 

The simulation results show the performance of the proposed protocol successfully prolongs the network lifetime and reduces energy consumption. The aim is to balance the energy consumption among the cluster members and cluster head. The clustering rotation strategy is based on the average energy threshold and average distance threshold and performs layering by the base station node. This can ensure more efficient energy consumption and network lifetime increment. On the other hand, we estimate a double cluster head to considerably reduce the overhead and energy consumption of the cluster head node. In this method, each cluster has two CH. The primary cluster head CH is responsible for aggregating data and forwarding it to the BS node if the distance of primary CH is greater than the distance threshold and the energy is less than the energy threshold. Meanwhile, the secondary cluster head 2CH is responsible for receiving and aggregating data within each cluster and sends the aggregated data to primary CH if the distance 2CH is less than the distance threshold and the energy of the 2CH is greater than the energy threshold.

**Figure 11:**
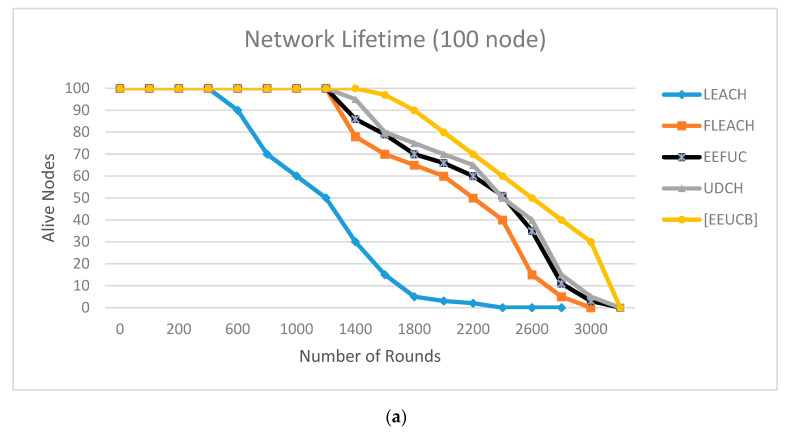

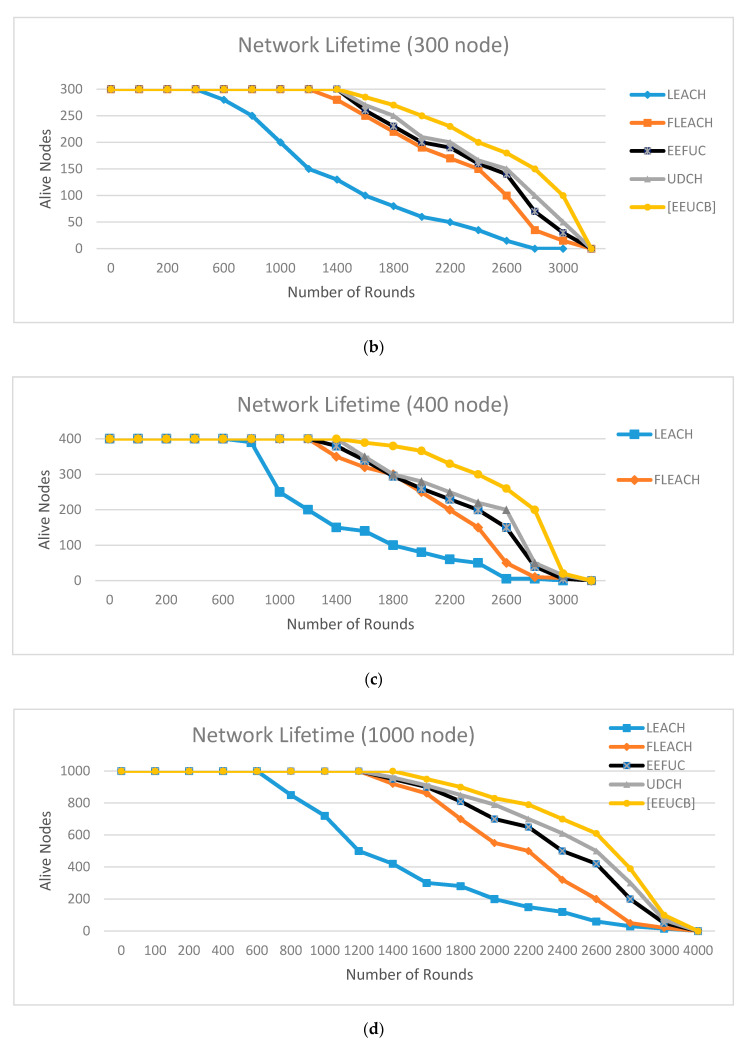
The network lifetime with (**a**) 100 nodes, (**b**) 300 nodes, (**c**) 400 nodes, (**d**) 1000 nodes.

**Figure 12:**
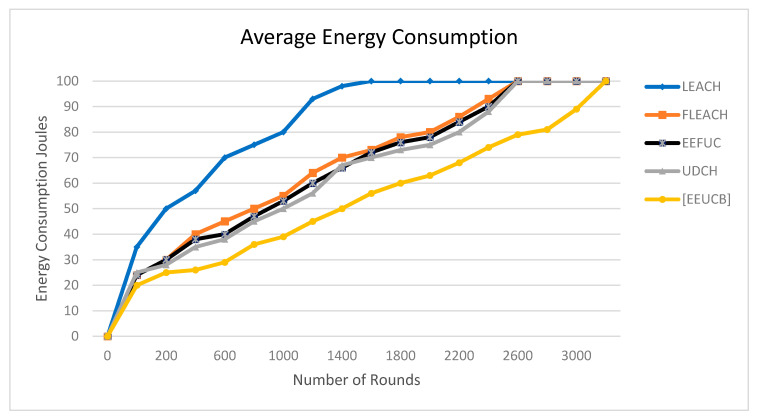
The average energy consumption.

**Figure 13:**
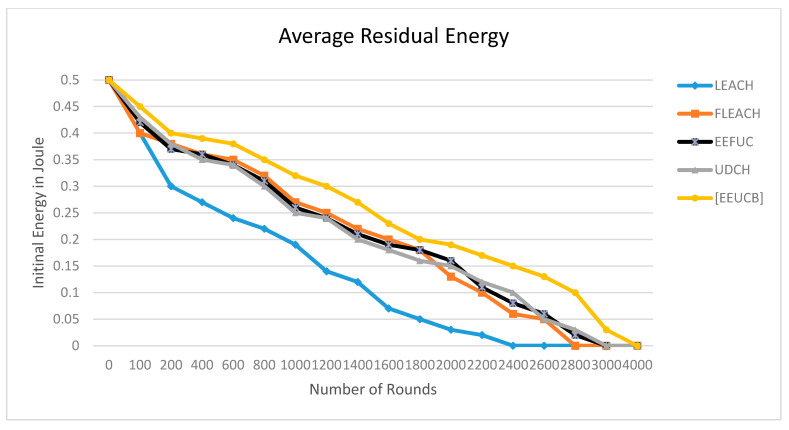
The average residual energy.

**Figure 14:**
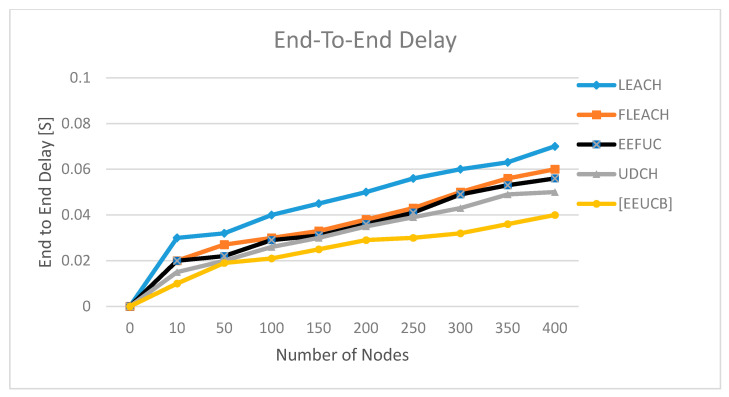
The end-to-end Delay.

**Figure 15:**
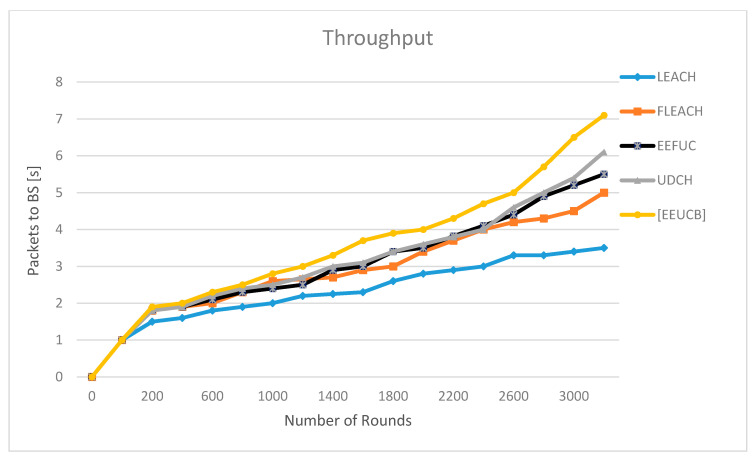
The throughput.

The performance comparison between the proposed protocol and the other three cluster protocols is presented in Figure 11, Figure 12, Figure 13, Figure 14 and Figure 15. 

Figure 11 plots the network lifetime, including the first node die (FND), half node die (HND), and the last node die (LND) between the proposed EEUCB, LEACH, FLEACH, EEFUC, and UDCH protocols in the first, second, third, and fourth scenarios is performed. From the figure, it can be observed that the proposed EEUCB protocol has increased the network lifetime in all four different scenarios. In the first of these, we deployed 100 sensor nodes in a 200 × 200$\;m^{2}$ area as shown in (Figure 11a). In (Figure 11b), we implemented the second scenario, the network area 300 × 300 ${\;m}^{2}$ with 300 sensor nodes. The third scenario, as shown in (Figure 11c), contains a 400 × 400 ${\;m}^{2}$ area, with 400 sensor nodes. Lastly, in (Figure 11d), we implemented the fourth scenario, the network area of 1000 × 1000 $m^{2}$ with 1000 sensor nodes, in order to check the scalability of the protocol. In addition, several different scenarios are generated to show the performance and to check the ability of the proposed protocol to reduce energy consumption and to prolong the network lifetime. 

Figure 11a shows the LEACH protocol FND is at 600 rounds and HND at 1150 rounds. In the FLEACH, EEFUC, and UDCH protocols, the FND is 1280, 1290, and 1301 rounds, and HND is at 2250, 2388, and 2390, respectively. In our EEUCB protocol, the FND is at 1420 rounds, and HND is at 2600 rounds. Figure 11b shows that the FND of LEACH, FLEACH, EEFUC, and UDCH protocols appear at 670, 1320, 1395, and 1421 rounds, respectively, while the HND appeared at 1176, 2320, 2400, and 2485, while in the EEUCB FND is at 1620 rounds, and HND at 2800. In Figure 11c, the LEACH, FLEACH, EEFUC, and UDCH protocols, the FND is at 695, 1322, 1402, and 1430 rounds, HND is at 1200, 2350, 2420, and 2500, and LND at 3000, 3320, 3600, and 3679. The FND of our EEUCB protocol stands at 1645 rounds, while HND and LND are at 2810 and 3800 rounds, respectively. In Figure 11d, the LEACH, FLEACH, EEFUC, and UDCH protocols, the FND is at 750, 1400, 1490, and 1525 rounds, HND is at 1300, 2398, 2500, and 2580 rounds, and LND at 3200, 3620, 3765, and 3800 rounds. The FND of our EEUCB protocol stands at 1700 rounds, while the HND and LND are at 2925 and 3920 rounds, respectively. 

The results show that our EEUCB protocol outperforms other protocols. The reason is that the proposed EEUCB protocol balances the energy consumption among the nodes in the network. This is achieved by an unequal clustering mechanism based on different competition radius calculation. The calculation of the competition radius for each node depends on a number of factors: the closest and farthest distance of the nodes from the BS, the distance from all the sensor nodes to the BS node, the residual energy of each node at each round, and the maximum capacity of the node energy compared to UDCH and the EEFUC protocol there are only calculating the distance form all nodes to BS, whereas the LEACH has not shown good performance in terms of the network lifetime because it did not propose an unequal clustering mechanism, instead the equal clustering mechanism was proposed. In addition, our EEUCB, UDCH, and FLEACH protocols utilize a double cluster head node in order to reduce the load on the primary CH. FLEACH has also not shown good performance in terms of the network lifetime due to the selection of the primary CH being randomly and alternately selected among the network nodes based on probability, whereas the selection of primary CH of EEUCB and UDCH is based on the minimum computing delay time of each node. However, EEUCB has shown an improved network lifetime because it utilizes the sleep-awake mechanism based on the distance from sensor nodes to CH and the energy level of the sensor nodes. The selection of 2CH of UDCH is based on the distance from the sensor nodes to the primary CH, so it has not shown an improved network lifetime, while our EEUCB protocol has shown an improved network lifetime because the selection of 2CH is based on calculating the highest residual energy of nodes, hence avoiding the selection of 2CH with low residual energy. Both the LEACH and EEFUC protocols have not shown an improved network lifetime because they only utilize one CH for aggregation and forward data transmission at the same time to the base station. 

On the other hand, the transmission round also helps to reduce the energy consumption of nodes by reducing the communication overhead and prolonging network lifetime in the network. As an example, if the location of the CHs is far from the BS, and the residual energy of the CHs is low, the CH may not be able to transmit data to the BS. This will lead to increases in energy consumption and packet loss during the data transmission in the network, and the CH might die sooner. The transmission round of EEUCB uses the average distance threshold and average energy threshold between CMs and CHs. Use the layer implementation and residual energy for the construct of a path to BS. Therefore, it has shown good performance in terms of the network lifetime compared to UDCH, FLEACH, LEACH, and EEFUC. The FLEACH protocol has not shown an improved network lifetime because it utilizes distance threshold only for transmission round between CMs and CHs, and to construct a path to the BS in the network. The transmission round between CMs and CHs, and to construct a path to the BS of EEFUC protocol utilize residual energy of sensor node and distance from CH to BS. The UDCH utilized between CMs and CHs uses the average energy threshold and the average energy to construct a path to BS in the network. 

In addition, we tested the statistical significance of the number of alive nodes (NOA) by using two-tailed T.TEST to draw a statistical inference as defined by [39,40]. A large sample consisting of pair of (NOA) in the proposed EEUCB protocol with other protocols such as LEACH, FLEACH, EEFUC, and UDCH are taken over different rounds behave like a normal co-related variable. Table 5 shows the results of two-tailed T.TEST for our proposed EEUCB protocol with other protocols. Our testing hypothesis has four cases that can be described as follows: 

Null Hypothesis $H_{0}$: ($NOA_{EEUCB}=NOA_{LEACH}).$ Alternative Hypothesis $H_{1}$: ($NOA_{EEUCB}>NOA_{LEACH}).$

Null Hypothesis $H_{0}$: ($NOA_{EEUCB}=NOA_{FLEACH}).$ Alternative Hypothesis $H_{1}$: ($NOA_{EEUCB}>NOA_{FLEACH}).$

Null Hypothesis $H_{0}$: ($NOA_{EEUCB}=NOA_{EEFUC}).$ Alternative Hypothesis $H_{1}$: ($NOA_{EEUCB}>NOA_{EEFUC}).$

Null Hypothesis $H_{0}$: ($NOA_{EEUCB}=NOA_{UDCH}).$ Alternative Hypothesis $H_{1}$: ($NOA_{EEUCB}>NOA_{UDCH}).$

The test statistic T with n-1 degrees of freedom can be computed as:

(19) $$T\;=\;D_{avg}÷\;(S_{d}÷\;\sqrt{\left(n-1\right))}$$

where $D_{avg}$ and $S_{d}$ denote the mean and standard deviation of the difference of NOA in two equal-sized correlated large samples of size n. The 95% confidence limits for $D_{avg}$.

(20) $$D_{avg}\;\pm \;T_{0.05}\times (S_{d}÷\;\sqrt{\left(n-1\right))}$$

where $T_{0.05}$ is the 5% point of t-distribution with n − 1 degrees of freedom. 

Let p indicate the probability of the calculated value for our statistical *t*-test with n-1 degrees of freedom to obey the null hypothesis. A value of *p* < 0.05 indicates that the null hypothesis is rejected at 5% significance level, and the alternative hypothesis be accepted at 95% confidence level. Our results obtained by *t*-test of our proposed EEUCB protocol with LEACH, FLEACH, EEFUC, UDCH protocols. In all the cases, *p* < 0.05, so the null hypothesis is rejected at 5 % significance level, and the alternative hypothesis is accepted at 95% confidence level. The lower and upper limits for the 95% confidence interval for $D_{avg}$ are shown in Table 5. Therefore, it can be observed that the proposed EEUCB protocol outperforms LEACH, FLEACH, EEFUC, and UDCH. 

Energy consumption is an essential factor in clustering protocols. Figure 12 plots the average energy consumption in joules as a function of the process number of rounds. The average energy consumption of our EEUCB protocol is less than the other four protocols, namely LEACH, FLEACH, EEFUC, and UDCH. The reason for this is because we improved the selection of CHs algorithms, the competition radius calculation, and transmission data operations, which leads to the distribution of CHs being more sensible and the reduction in energy consumption of the sensor nodes. By contrast, the UDCH calculated the computation radius and the distance from nodes to the BS but did not calculate the maximum capacity of the node energy in leading to an increase in the energy consumption of nodes. The LEACH has larger results than other protocols due to the cluster distributed randomly in the network and did not utilize a double cluster head. Therefore, it increases the load on the main CH, while the FLEACH utilizes a double cluster head but did not address the hot spots problem and randomly selection primary CH, which leads to the consumption of more energy and unbalanced energy consumption for receiving and transmitting data to the BS. The EEFUC addressed the hot spots problem but did not utilize a double cluster head, therefore, increasing the load on the main CH in the network. 

In addition, we tested the statistical significance of the energy consumption for a single round using a pair-wise *t*-test to draw a statistical inference as defined as shown in Table 6. The calculation of the *t*-test of the energy consumption process is the same as the *t*-test of (NOA), as in the Equations (19) and (20). In all the cases *p* < 0.05, so the null hypothesis is rejected at 5 % significance level, and the alternative hypothesis is accepted at 95% confidence level. Furthermore, we checked the impact of the network capacity of energy consumption per round by testing our protocol with different scenarios are shown in Table 7. 

The average residual energy is calculated from the remaining energy after the packet’s transmission process between sensor nodes. Figure 13 shows the energy balance evaluation of EEUCB. To obtain an accurate evaluation, we run the simulation ten times, and average results are computed. Figure 13 shows the average residual energy consumption comparison of our proposed EEUCB protocol with four protocols; LEACH, FLEACH, EEFUC, and UDCH. From the result, the average residual energy of the four protocols is less than the proposed EEUCB protocol. The average residual energy starts to drop below the half initial energy from 1000 rounds in LEACH and FLEACH, and in EEFUC and UDCH from 1200 rounds. The residual energy of the proposed EEUCB protocol, on the other hand, only starts to drop at 1400 rounds. Table 8 presents the standard deviation of residual energy against a different number of rounds. Results show that our EEUCB protocol outperforms other protocols and that EEUCB has a better energy balance compared to other protocols. The EEUCB protocol reduces energy consumption by reducing the communication overhead and utilizes the sleep-awake mechanism based on the distance from sensor nodes to the CH and the energy level of the sensor nodes. The selection of CH methods used in the proposed EEUCB protocol enhances load balancing based on minimum delay time with the sleep-awake mechanism and the highest residual energy methods. In contrast, in UDCH, the selection of the CHs process focuses on the delay time for the primary CH and the distance from the nodes to the CH. This could lead to the selection of CH nodes with low residual energy that may die early. 

The selection of CH in LEACH FLEACH, and EEFUC are randomly selected based on probability, which leads to consuming more energy for the selection and transmission process. In addition, the transmission process is very important in clustering protocols due to more dissipated energy in the transmission process. Therefore, EEUCB has good results because we enhance the transmission process based on the energy and distance thresholds between the CMs and CHs and the layering implementation methods between the CHs and the BS node. For example, if the distance from the primary CH is less than the distance threshold and the residual energy is greater than the energy threshold, the nodes will send data directly via single-hop to the primary CH; the primary CH then receives the data and applies aggregate functions. These nodes help the CH to consume less energy in receiving data. However, if the distance from the primary CH is greater than the distance threshold, and the residual energy is less than the energy threshold, the system will use multi-hop routing to transfer data to the CH, which consumes more energy. On this basis, the nodes will send data to the 2CH, which will aggregate data to be sent to the primary CH, whereas the transmission between CHs and BS node beads on the layering process. If the CH [i] is not located in the first layer (not close to the BS), and the residual energy of the CH [i] is less than the residual energy of CH [j], the CH [i] will send aggregated data to the CH [j], and the CH [j] will transmit the data to BS; otherwise, the CH [i] transmit aggregated data to the BS directly. Therefore, these methods helped us to preserve energy consumption in the network. The transmission process between the CMs, CHs, and BS was based on the distance threshold in FLEACH. The distance threshold was perhaps insufficient to preserve the residual energy of nodes, which leads to a loss of residual energy for nodes. In EEFUC, the transmission was based on residual energy; however, the CH consumed more energy. Hence, it did not save much residual energy in sensor nodes, whereas UDCH uses only the average energy threshold for the transmission process. 

Figure 14 shows the end-to-end delay results (in seconds). The end-to-end delay can be defined as the packets transfer from sensor nodes to the sink node. The maximum delay of our proposed EEUCB protocol was recorded at 0.04 seconds, whereas the prior protocols have higher latency between 0.05–0.07. The lower delay time obtained by the proposed EEUCB is due to the inclusion of distance information between the sensor node to the base station by calculating the minimum and maximum distance and also using a double cluster head in each cluster. The distance information helps in terms of the layers’ implementation method to estimate the distance between cluster nodes to the BS and dividing into four layers based on the furthest and closest distance to BS. The FLEACH and UDCH used a double cluster head without calculating the minimum and maximum distance. Therefore, the location of CHs may be furthest from the BS, generating a delay and increasing energy consumption, whereas the LEACH and EEFUC do not propose a double cluster head nor calculate the minimum and maximum distance in the network. 

Figure 15 shows the throughput results, which can be defined as the number of data packets received at the BS in a period of time. We have tested the throughput with the 1000 number of nodes. Results show that the throughput of our proposed EEUCB protocol performed better than the other four protocols. In LEACH, the received data packets are about 350,000, FLEACH about 520,000, EEFUC about 550,000, and UDCH around 610,000, while the EEUCB received data packets at about 700,000. Table 9 shows the results of a paired *t*-test for our proposed EEUCB protocol with other protocols. The calculation of the *t*-test of the throughput process is the same as the *t*-test of (NOA), as in the Equations (19) and (20). A value of *p* < 0.05 indicates that the null hypothesis be rejected at 5% significance level, and the alternative be accepted at a 95% confidence level. In every case where *p* < 0.05 the null hypothesis is rejected at 5% significance level and the alternative hypothesis is accepted at 95% confidence level. The proposed EEUCB managed to obtain the highest throughput because it used the sleep-awake mechanism among nodes in the network. Therefore, the redundant data transmission among nodes is limited, restricted contrary to UDCH and EEFUC. Moreover, the EEUCB used the layering implementation method between CHs and BS. The LEACH has the lowest throughput because it did not consider the unequal clustering and sleep-awake mechanism. The proposed EEUCB protocol has shown an increase in throughput to the BS over other protocols. 

## 6. Discussion 

The proposed EEUCB protocol is an improved version of the UDCH protocol. Both methods utilize the delay time method to select the CH in the network. However, further improvements are made in EEUCB by considering the sleep-awake mechanism. In addition, the proposed EEUCB protocol takes into account the highest residual energy for the selection of the 2CH and the distance between two nodes to check the node ID, location, neighbor nodes, and the number of neighbor nodes. In addition, the distance of cluster nodes is distributed in the network by layering calculations. In our protocol, the BS calculates the distance based on the closest and farthest nodes and divides this distance into four layers. The advantage of this method is that the nodes in the first layer can send the data to the BS through a single hop. If the nodes are in the second, third, or fourth layer, it will send the data to the CH through a multi-hop. This method helps to reduce and maintain energy consumption in a network. Besides that, the sleep-awake mode is utilized to maintain the energy of the node and to prolong the lifetime of the network. This is due to the fact that the active nodes furthest from the CH will reduce energy efficiency and will die early.

On the other hand, the selection of non-CHs is dependent on the minimum distance between non-CHs and the CH because the closest distance between the CM and the CH will not dissipate more energy. At the same time, the node can send data during long rounds rather than transmitting with a longer distance, which requires more energy and time. In addition, our protocol utilizes the energy and distance threshold to balance energy consumption within nodes; this allows the two CHs to be selected to reduce the overhead on the primary CH and to enable them to distribute the operations between them. The layering implementation and residual energy construct the path to the BS. These methods are proposed for the transmission of data from the CH to the BS node in a scenario where the primary CH is situated far from the BS node and is not located in the first layer. This will increase energy consumption and overhead. In order to resolve this, the primary CH will send the data to the other CH that is closest to the BS node. The comparison between our method and prior methods is listed in Table 10. 

## 7. Conclusions 

In this paper, we investigate energy efficiency and solutions to the hot spots problem by proposing a method called EEUCB. Unlike prior unequal clustering methods such as UDCH, our protocol utilizes an unequal clustering mechanism based on a different competition radius calculation. The calculation of the competition radius for each node depends on a few factors: (i) the closest and farthest distance of the nodes from the BS; (ii) the distance from all the sensor nodes to the BS node; (iii) the residual energy of each node at each round; and (iv) the maximum capacity of the node energy. The utilization of the distance between nodes and the BS reduces and maintains energy consumption in a network. 

Other than unequal clustering, the proposed EEUCB protocol also considers a double cluster head implementation. The EEUCB is different from the prior double CHs implementation in terms of the selection of 2CHs based on the highest residual energy of nodes. This would reduce the overheads on the primary CH and enable them to distribute the operations between them. 

Moreover, to balance the energy consumption among CMs and the CH, a clustering rotation strategy based on a few factors is proposed, namely the average energy threshold, average distance threshold, and the use of the layering implementation to construct the path to the BS. This increases the efficiency of energy consumption and the network lifetime. The results of our proposed EEUCB protocol show lifetime improvements of 57.75%, 19.75%, 14.7%, and 13.06% against LEACH, FLEACH, EEFUC, and UDCH, respectively. In future works, we will optimize our work by using software-defined networking (SDN) to improve WSN energy efficiency.

## Figures and Tables

**Table 1:** Summary of existing works.

Ref.	Description	Contribution	Limitation
Low energy adaptive clustering hierarchy (LEACH) [22]	In LEACH, the selection of cluster head (CH) is randomly based on probability between nodes, and the base station is randomized rotationally. The responsibility of CH is to collect data from cluster members, perform data aggregation, and forward them to the base station directly.	Reduces communication between the nodes and base station in order to preserve energy in the network. Utilizes data aggregation technique in order to reduce the data redundancy transmission.	The LEACH does not address the hot spots problem. The randomly selected cluster head leads to loss of energy, and the nodes are terminated sooner.
Hybrid energy-efficient distributed clustering (HEED) [23]	In HEED, the selection of CH was based on the remaining energy of nodes and the communication cost.	Multi-hop routing Inter-cluster and intra-cluster transmission were also used.	The overhead of the HEED protocol was high.
Low energy adaptive tier clustering hierarchical routing (LEATCH) [24]	A two-level hierarchical clustering approach is proposed to guarantee communication between cluster nodes and the base station in the network. The selection of CH is randomly based on probability between nodes, and the base station is randomized rotationally.	The advantage of this protocol was reduced delay and energy consumption.	The balanced energy consumption technique between cluster nodes was not addressed.
Modified low energy adaptive clustering hierarchy (LEACH-M) [25]	The selection of CH was based on residual energy, and the location address of nodes in the network was proposed. The optimization of the CHs threshold equation is based on the network address of nodes and residual energy.	This protocol successfully balanced the network energy burden and dramatically improved energy efficiency.	The distance among nodes and the sleep and awake mode was not considered and leads to increases in energy consumption in the network.
Distributed energy-efficient clustering (DEEC) [26]	In DEEC, proposed protocol for heterogeneous WSN. The selection of CH was based on a ratio of residual energy of each node and the average energy level of the network.	The advantage of this protocol was that it improved energy efficiency in the network.	It does not address the close distance between cluster nodes and the base station and leads to the hot spots problem when more than one cluster transmitting data to BS.
Energy-efficient unequal clustering mechanism (EEUC) [27]	In EEUC, the selection of CH was based on localized computation and also utilized the unequal clustering technique on nodes in the network. In addition, proposed multi-hop routing for the inter-cluster communication, the CH elect a relay node from it close to CHs according to the distance and residual energy of nodes to BS.	Decreased the distance between nodes and the base station whereby was divided the network into unequal clustering sizes.	Elected one CH for aggregation and forward data transmission at the same time to the base station.
Energy-efficient Multi-hop Unequal Clustering method (EMUC) [28]	In EMUC, the selection of CHs was based on the residual energy of nodes. EMUC uses the Dijkstra routing algorithm to create the shortest path between the nodes and CHs.	Reduce energy consumption by performed inter-cluster communication in multi-hop nodes.	This method did not reduce the load on the cluster head node and was not applied to the sleep-awake mechanism, which led to increasing the energy consumption in the network.
Improved energy-aware distributed unequal clustering protocol (EADUC) [29]	In EADUC, the proposed protocol to solve the hot spots problem. The selection of CHs via information and numbering of neighbor nodes and the location of BS and residual energy is given as clustering parameters. The selection of relay nodes is based on the cost and energy of nodes instead of only the distance information.	Balanced energy consumption and increased throughput.	Non-applied sleep-awake mechanism.
Fuzzy energy-aware unequal clustering algorithm (EAUCF) [30]	In EAUCF, the proposed method to prolong the network lifetime. The proposed probabilistic model for selecting CH is based on the residual energy of nodes. The competition ranges in this method use the distance and residual energy of nodes to BS.	This protocol addressed the hot spots problem in the network.	The balanced energy consumption among nodes was not addressed.
Balanced-Imbalanced Cluster Algorithm (B-IBCA) [31]	In B-IBCA, proposed Stabilized Boltzmann Approach (SBA) for unequal clustering. The selection of CH was based on the distance and residual energy of nodes to the base station and used the Boltzmann model to check the energy consumption of nodes and determine the priority of nodes to CHs.	This protocol addressed the hot spots problem in the network.	This method did not reduce the load on the cluster head node and was not applied to the sleep-awake mechanism, which led to an increase in energy consumption in the network.
Two-tier distributed fuzzy logic-based protocol (TTDFP) [32]	Proposed fuzzy logic in order to increase network lifetime and to address the data aggregation problems of multi-hop. The selection of CH is based on the distance nodes to BS. This method uses two fuzzy parameters, relative distance (RD) and average link residual energy (ALRE), to determine the best routing path for transmission between nodes and CHs.	Addressed the hot spots problem and calculated the distance to BS.	This method did not reduce the load on the cluster head node and was not applied to sleep-wake mechanism, which led to increasing the energy consumption in the network.
Energy-efficient cluster head selection scheme (EECHS) [33]	In EECHS, one node in each cluster is selected to monitor the energy of all sensor nodes and CHs called scheduling nodes. The CH is randomly and alternately selected among the network nodes based on probability. The transmission process between sensor nodes and CHs dynamically to BS according to the residual energy of nodes.	Reduced the delay transmission data in the network.	Unbalanced energy consumption in the network.
Fuzzy-based unequal clustering (FBUC) [15]	In FBUC, the CHs are elected in the network by energy level, and CMs join the CHs based on a fuzzy system with distance from CH, and CH numbers are inputs of the fuzzy system. The non-CH nodes join the CH based on the distance from nodes to CH.	Reduced the transmission delay in the network.	Increased overhead in the network.
Energy conserved unequal clusters with Fuzzy logic (ECUCF) [34]	In ECUCF, the selection of CH is randomly based on probability between nodes, and the base station is randomized rotationally.	Addressed the hot spots problem in the network.	Utilizes one cluster head for aggregation and data transmission to the base station
Distributed Clustering Algorithm Guided by the Base Station (DCAGBS) [35]	In DCAGBS, the selection of CHs was based on a fuzzy-logic system. In particular, the messages of the BS are used to tune the skip value dynamically.	Reduced CHs selection overhead.	The balanced energy consumption and the hot spots problem was not addressed.
Multi- clustering algorithm using fuzzy logic (MCFL) [36]	Utilized fuzzy and non-fuzzy in order to select CH, clustering nodes in different rounds use different clustering algorithms.	Increased throughput by increasing the number of messages addressed to the base station.	The hot spots problem was not addressed in this method.
Energy-efficient unequal clustering routing protocol for wireless sensor networks (UDCH) [4]	In UDCH, proposed unequal clustering technology to solve the hot spots problem and double CHs to reduce energy consumption. In addition, they also proposed a hybrid rotation strategy to reduce also energy consumption based on the time and energy of nodes.	They addressed the hot spots problem in the network.	The distance threshold among nodes was not calculated, which led to the reduction of network lifetime and increased energy consumption. Also, the sleep-awake mechanism among nodes was not employed
Factor-based LEACH (FLEACH) [18]	In FLEACH, the random selection of CH was based on probability. Selection of multi-level CH in order to reduce the load on primary CH.	The secondary cluster head was determined based on the highest residual energy of the nodes.	The hot spots problem was not addressed.
Energy-efficient fuzzy logic for unequal clustering (EEFUC) [19]	In EEFUC, a fuzzy logic method was utilized in order to reduce energy consumption and multi-hop clustering in the network. The selection of CHs was based on the distance to BS, current energy of nodes, and the number of live nodes in the network.	The selection of multi-hop clustering was based on higher residual energy.	The balanced energy consumption among nodes was not addressed, which led to an increase in communication overhead in the network.
Multi-objective fuzzy clustering algorithm (MOFCA) [37]	In MOFCA, the proposed method in order to balance energy consumption and to solve the hot spots problem. Uses competition radius via energy for selecting the final CHs.	Balanced energy consumption among nodes in the network.	The load on the cluster head was not considered, which led to the consumption of more energy for transmission data. Also, the sleep-awake mechanism was not applied.

**Table 2:** Notation and description.

Notations	Description	Notations	Description
a, b	Is the weight factor	$E_{rem}$	Is the average residual energy of nodes
**σ**	Is a tiny number	$E_{rem}$ *(**i**, **r**)*	The residual energy of node i at round r
**$avg_{D}$**	Is the average distance	$E_{th}$	Is the energy threshold
BS	Base station	***j***	The neighbor node of node i
CH	Cluster head node	k	Is the length of transmission data
**$d_{0}$**	Threshold distance value	**n**	Is the variable increment
**$D_{th}$**	Is the distance threshold	**N**	Is the total number of sensor nodes
**$D_{t}$**	The delay time of node i	NN(i,r)	The set of neighbor nodes
**$d_{L}$**	Distance length of each layer	⋮NN(i,r)⋮	Is the number of neighbors
**$D_{max}$**	Is the maximum distance from nodes to BS	$P_{t\left(i\right)}$	Is the time when sending packets
**$d_{max}$**	Is the farthest distance of the node from the BS	$P_{r\left(i\right)}$	Is the time when packets are received
**$d_{min}$**	Is the closest distance of a node from the BS	**r**	Is the number of rounds
**$D_{itoBS}$**	The distance of each node to the BS	***R***	Is the radius
**$d_{i,BS}$**	Is the distance from node i to BS	$R_{c}\left(i\right)$	Is the radius of node i
**efs**	Is the data energy consumption of 1-bit in free space mode	$RL_{max}$	The maximum competition radius for becoming CH
**emp**	is the data energy consumption of a 1-bit in multi-path attention mode	$R_{v}$	Is the random value
$E_{init}$ *(i)*	The initial energy of node i	$S_{d}$	The standard deviation
**$E_{CH}$**	Is the initial energy of CH	$T_{CH}$	Is the total number of CH in the network
**$E_{elec}$**	Is the energy consumption of the receiver circuit or sender circuit for 1-bit data	$T_{E}$	Is the total energy of sensor nodes in the cluster
**$E_{max}$**	Maximum capacity of node energy	$Th_{v}$	The threshold value of the node in the cluster
**$E_{TX}$**	Is the energy consumption of the transmitter	$T_{p}$	Is the throughput
**$E_{RX}$**	Is the energy consumption of the receiver	$T_{r}$	Is the total number of packets received at BS
**$E_{avg\left(i\right)}$**	The average energy of neighboring nodes i	$W_{t}$	The computation time of primary CH
**$E_{avg}$**	Is the average energy consumption	Z	Is the name of each node
$E_{rem}$ *(i)*	Is the residual energy of node i	∂,β	Is the weight factor in determining the impact of the distance between the CMs and CHs

**Table 3:** Scenarios for the proposed protocol.

Scenario	Number of Sensor Nodes	Network Space
Scenario_1	100	200 × 200 m^2^
Scenario_2	300	300 × 300 m^2^
Scenario_3	400	400 × 400 m^2^
Scenario_4	1000	1000 × 1000 m^2^

**Table 4:** Parameters of simulations.

Parameters in EEUCB	Value
Sensing area	200 × 200 m^2^ (Scenario_1) 300 × 300 m^2^ (Scenario_2) 400 × 400 m^2^ (Scenario_3) 1000 × 1000 m^2^ (Scenario_4)
Number of nodes	100, 300, 400, 1000
The initial energy of sensor nodes	0.5 joules
Data packet size	4000 bits
Control message size	200 bits
Maximum communication radius $RL_{max}$	70 m
Waiting time, $W_{t}$	5 s
Transmission energy, efs	10 pJ/bit/$m^{2}$
Transmission energy (long-distance emp)	0.0013 pJ/bit/$m^{4}$
Electronic circuit energy, $E_{elec}$	50 pJ/bit
Aggregation energy	5 pJ/bit

**Table 5:** Results of *t*-test.

[EEUCB]	*t*-Test	Significance of the Null Hypothesis	Confidence Interval 95%	
			Lower	Upper
LEACH	47.21	<5%	35.37	38.63
FLEACH	21.19	<5%	9.6	17.24
EEFUC	17.69	<5%	5.33	13.66
UDCH	13.46	<5%	3.64	10.58

**Table 6:** Results of *t*-test of energy consumption for a single round.

[EEUCB]	*t*-Test	Significance of the Null Hypothesis	Confidence Interval 95%	
			Lower	Upper
LEACH	12.68	<5%	12.66	12.71
FLEACH	7.96	<5%	7.91	8.033
EEFUC	4.13	<5%	4.11	4.14
UDCH	2.37	<5%	1.99	2.38

**Table 7:** The energy consumption with different scenarios.

Network Capacity	LEACH	FLEACH	EEFUC	UDCH	[EEUCB]
Scenario_1	0.0495	0.0397	0.0298	0.0294	0.014
Scenario_2	0.0582	0.0412	0.0284	0.0226	0.0196
Scenario_3	0.0532	0.0434	0.0356	0.0297	0.0135
Scenario_4	0.0576	0.0422	0.0284	0.0221	0.0179

**Table 8:** Standard deviation residual energy.

No. Rounds	LEACH	FLEACH	EEFUC	UDCH	[EEUCB]
**1000**	0.413502	0.273344	0.252321	0.229902	0.143445
**2000**	0.426451	0.282043	0.255009	0.232022	0.146405
**3000**	0.427665	0.286485	0.261123	0.238551	0.149894
**4000**	0.431013	0.288559	0.264544	0.242256	0.153445

**Table 9:** Results of *t*-test for throughput.

[EEUCB]	*t*-Test	Significance of the Null Hypothesis	Confidence Interval 95%	
			Lower	Upper
LEACH	33.24	<5%	24.46	37.80
FLEACH	18.55	<5%	14.30	7.60
EEFUC	14.42	<5%	10.74	12.90
UDCH	8.66	<5%	2.55	5.16

**Table 10:** Comparison between our method and prior methods.

FLEACH	EEFUC	UDCH	EEUCB (Proposed Protocol)
The placement of the sensor does not depend on the network layer.	The placement of the sensor does not depend on the network layer.	The placement of the sensor does not depend on the network layer.	The placement of the sensor nodes based on the network layer as in Algorithm 1.
Does not propose an unequal clustering mechanism. The equal clustering mechanism was proposed instead.	Proposes an unequal clustering mechanism based on competition radius: The calculation of the competition radius for each node depends on: The residual energy of sensor nodes. The distance $d_{i,BS}$ from all the sensor nodes to the base station node.	Proposes an unequal clustering mechanism based on the competition radius $R_{c}\left(i\right)\;\mathrm{such}\;\mathrm{as}\;\mathrm{in}\;\mathrm{Equation}\;\left(5\right).$ The calculation of the competition radius for each node depends on: The residual energy of sensor nodes $E_{rem}\left(i\right)$. $\mathrm{The}\;\mathrm{distance}\;d_{i,BS}$ from all the sensor nodes to the base station node.	Propose unequal clustering mechanism based on the competition radius $R_{c}\left(i\right)\;\mathrm{such}\;\mathrm{as}\;\mathrm{in}\;\mathrm{Equation}\;\left(6\right)$. The calculation of the competition radius for each node depends on: The residual energy of each node at each round $\;E_{rem}\left(i,r\right)$. The distance $d_{i,BS}$ from all the sensor nodes to the base station node. $\mathrm{The}\;\mathrm{minimum}\;\mathrm{distance}\;d_{\min}$ of the closest node from the base station. $\mathrm{The}\;\mathrm{maximum}\;\mathrm{distance}\;d_{\max}$ of the furthest node from the base station. The maximum capacity of node energy $E_{max}$.
FLEACH utilizes a double cluster head node in order to reduce the load on primary CH. The selection of CHs is as follows: During the cluster formation phase, a CH is randomly and alternately selected among the network nodes based on probability.	EEFUC utilizes one CH for aggregation and forwards data transmission at the same time to the base station. During the cluster formation phase, a CH is randomly and alternately selected among the network nodes based on probability.	UDCH utilizes a double cluster head node in order to reduce the load on primary CH. The selection of CHs is as follows: The selection of primary CH is based on the minimum computing delay time $D_{t}\left(i\right)\;$ of each node. The selection of 2CH is based on the distance from the sensor nodes to the primary CH.	Our EEUCB utilizes a double cluster head node in order to reduce the load on primary CH. The selection of CHs is as follows: The primary CH selection method for the proposed EEUCB is similar to the method in UDCH. However, in EEUCB, further improvement is done by considering the sleep-awake mechanism based on the distance from sensor nodes to CH, and the energy level of sensor nodes. The selection of 2CH is based on calculating the highest residual energy of nodes.
Transmission round between CMs and CHs use distance threshold $D_{th}$ and use the distance threshold to construct a path to BS in the network.	Transmission round between CMs and CHs, and to construct a path to BS in the network, use residual energy of sensor node and distance from CH to BS.	Transmission round between CMs and CHs use the average energy threshold $E_{th}$ and use the average energy to construct a path to BS in the network.	$\mathrm{Transmission}\;\mathrm{round}\;\mathrm{between}\;\mathrm{CMs}\;\mathrm{and}\;\mathrm{CHs}\;\mathrm{use}\;\mathrm{average}\;\mathrm{distance}\;\mathrm{threshold}\;D_{th}$, average energy threshold $E_{th}$. Use the layer implementation and residual energy for the construct of a path to BS.

## Data Availability

Not applicable.
